# A novel Monoclonal Antibody against Notch1 Targets Leukemia-associated Mutant Notch1 and Depletes Therapy Resistant Cancer Stem Cells in Solid Tumors

**DOI:** 10.1038/srep11012

**Published:** 2015-06-05

**Authors:** Ankur Sharma, Rupali A Gadkari, Satthenapalli V Ramakanth, Krishnanand Padmanabhan, Davanam S Madhumathi, Lakshmi Devi, Lingappa Appaji, Jon C Aster, Annapoorni Rangarajan, Rajan R Dighe

**Affiliations:** 1 Department of Molecular Reproduction Development and Genetics, Indian Institute of Science Bangalore, Karnataka, India; 2 Molecular Biophysics Unit, Indian Institute of Science Bangalore, Karnataka, India; 3 Department of Pathology, Kidwai Memorial Institute of Oncology, Bangalore, Karnataka, India; 4 Department of Pediatric Oncology, Kidwai Memorial Institute of Oncology, Bangalore, Karnataka, India; 5 Department of Pathology, Brigham & Women’s Hospital, Harvard Medical School, Boston, USA

## Abstract

Higher Notch signaling is known to be associated with hematological and solid cancers. We developed a potential immunotherapeutic monoclonal antibody (MAb) specific for the Negative Regulatory Region of Notch1 (NRR). The MAb604.107 exhibited higher affinity for the “Gain-of-function” mutants of Notch1 NRR associated with T Acute lymphoblastic Leukemia (T-ALL). Modeling of the mutant NRR with 12 amino-acid insertion demonstrated “opening” resulting in exposure of the S2-cleavage site leading to activated Notch1 signaling. The MAb, at low concentrations (1–2 μg/ml), inhibited elevated ligand-independent Notch1 signaling of NRR mutants, augmented effect of Thapsigargin, an inhibitor of mutant Notch1, but had no effect on the wild-type Notch1. The antibody decreased proliferation of the primary T-ALL cells and depleted leukemia initiating CD34/CD44 high population. At relatively high concentrations, (10–20 μg/ml), the MAb affected Notch1 signaling in the breast and colon cancer cell lines. The Notch-high cells sorted from solid-tumor cell lines exhibited characteristics of cancer stem cells, which were inhibited by the MAb. The antibody also increased the sensitivity to Doxorubucinirubicin. Further, the MAb impeded the growth of xenografts from breast and colon cancer cells potentiated regression of the tumors along with Doxorubucin. Thus, this antibody is potential immunotherapeutic tool for different cancers.

The Notch signaling is an evolutionarily conserved pathway involved in various cellular processes such as maintenance of stem cells and adult homeostasis^1^. Notch receptor-ligand interactions bring about conformational changes in the Negative Regulatory Region (NRR) followed by a series of proteolytic events (S2 and S3) catalyzed by ADAM/TACE metalloproteases and γ-secretase^2^. Once released, the Notch intracellular domain (N-ICD) translocates to the nucleus and associates with the DNA binding proteins leading to an active transcription complex that in turn activates the downstream signaling cascade in a context-dependent manner^3^. Aberrant Notch signaling has been associated with several developmental disorders and certain cancers^4^. Over expression of Notch receptors and ligands has been associated with solid tumors while ‘gain-of-function’ mutations are more frequent in hematological malignancies^5^,^6^.

Recent evidence suggests existence of long-term, self-renewing ‘tumor initiating cells’ or ‘cancer stem cells’ (CSCs) in various cancers^7^. The CSCs are inherently chemotherapy resistant cells and may lead to tumor relapse^8^. The Notch signaling pathway plays an important role in the maintenance of these CSC sub-populations and also contributes to chemotherapy resistance^9^,^10^. Hence, targeting the Notch signaling pathway provides an attractive opportunity for specific targeting of CSCs. Various strategies are being developed to block Notch signaling in cancer cells, the most prominent being inhibition of proteolytic cleavage by γ-secretase inhibitors (GSIs)^11^. However, GSIs, in addition to being pan-Notch inhibitors also block the processing of many other transmembrane proteins and must be given intermittently due to dose-limiting on-Notch toxicities^12^,^13^,^14^,^15^. In principle, specific monoclonal antibodies that distinguish among the paralogous receptors can overcome both these limitations of GSIs. Recent studies have demonstrated success of such paralogue-specific anti-Notch antibodies in therapeutic targeting of various cancers^9^,^16^,^17^,^18^. Earlier data from our laboratory have demonstrated the effectiveness of MAbs against the ligand-binding domain of Notch1 in therapeutic targeting of breast cancer stem-like cells^17^.

Acquired ‘gain-of-function’ mutations in Notch1 have been reported in 40–50% of T-cell acute lymphoblastic leukemias (T-ALL)^19^. These mutations induce conformational changes in the NRR and disengage the heterodimerization domain (HD) leading to ligand-independent receptor activation^20^. Despite several claims of successful antibody-mediated therapeutic targeting of Notch1, specific MAbs recognizing the NRR mutants have not been reported. In the present study, we report a MAb against the NRR of Notch1 that recognizes the “Gain of Function” mutant receptors with relatively higher affinities. This MAb can deplete Leukemia Initiating cells in the T-ALL cells and can also effectively target the chemotherapy-resistant CSCs in breast and colon cell lines and impede tumor progression *in vivo* clearly indicating its therapeutic potential.

## Experimental procedures

### Generation and characterization of Notch1 receptor fragments

Human Notch1 NRR (amino acid 1448–1725) was expressed as GST-fusion protein as described previously^21^. The Lin-12 Notch Repeats (LNR) of Notch1 (LNR-A, LNR-B, LNR-C) and the HD domain were amplified using specific primers and Notch1 cDNA as the template. The mutant Notch1 NRR fragments (L1594P, R1599P and I1681N) were amplified from the full-length Notch1 cDNAs bearing these mutations^22^ as the template and expressed as GST-fusion proteins and further purified using GSH affinity chromatography.

### Cell lines

The HEK293 cell lines stably overexpressing human Notch1 (HEK-hN1) and human Notch2 (HEK-hN2) were described previously^17^. The cancer cell lines MCF-7, BT-474, MDA-MB-231, HCC-1806, and HCT-116 were obtained from ATCC while Jurkat and CCRF-CEM was procured from NCCS, Pune, India and maintained under prescribed growth conditions. Generation and characterization of Jagged-1Fc has been described previously^17^.

### Structure analysis

#### Molecular modeling

Using Modeller^23^, a 3D structural model was generated for the mutant Notch1 harboring 12 amino acid insertion (19) using the wild type Notch1 (PDB id:3ETO)^24^ as template. The generated model was energy minimized to avoid any short contacts. The structures of wild type and mutant NRRs were then superimposed and visualized using Pymol software^25^.

#### Interface determination

The generated structural model of the mutant was subsequently used to determine the domain-domain interaction interface residues^26^. The interface residues were determined using distance criterion wherein a residue pair, with two residues from different domains, is said to be interacting if the distance between the two is less than or equal to the sum of their van der Waals radii plus 0.5 A°. The interface residues determined were then compared to those in the wild type structure.

### Generation and characterization of monoclonal antibodies

All *in vivo* experiments were performed in accordance with the relevant guidelines and regulations with prior approval from the Animal Ethics Committee of the Indian Institute of Science, Bangalore). Mice were immunized with purified NRR-GST fusion protein and MAbs were generated using the protocols established for the glycoprotein hormones^17^. Binding of MAbs to the wild type and mutant Notch1 NRR proteins (L1594P, R1599P and I1681N) as well as other sub-domains (LNR-A, LNR-B, LNR-C and HD) was determined using standard ELISA protocols.

### Flow cytometry assay

The cells were harvested using DPBS-EDTA, resuspended in DPBS containing 2% fetal bovine serum (FBS; Invitrogen, USA) (FBS/PBS) and binding of anti-Notch1 antibodies to the full-length Notch1 receptor was determined using the Becton Dickinson FACSCanto as described previously^17^,^21^. Ability of MAbs to distinguish between the wild type and mutant Notch1 receptor was determined by calculating the median fluorescence intensities using the ‘Stat’ program of “CellQuest” from Becton Dickinson.

### Surface plasmon resonance

The wild type and mutant (L1594P) Notch1 NRR proteins were dissolved in 50 mM sodium acetate buffer, pH 5.5, and immobilized on an EDC/NHS[N-ethyl-N’-(3-dimethyl amino propyl) – carbo-di-imide-hydrochloride/ (N-hydroxy succinimide)] -activated CM5 sensor chip as recommended by the manufacturer (BIAcore AB) yielding a surface density of approximately 1000 resonance units. MAbs 604.107, 604.132 and 604.164 were diluted in 10 mM HEPES, 0.15 M NaCl, 3.4 mM EDTA, 0.05% surfactant P-20 binding buffer, pH 7.4 (HBS) and allowed to bind at 25 °C using a flow-rate of 10 μl/min as previously described^27^. The dissociation constant, K_D_, was calculated from the ratio of dissociation rate (K_OFF_) to association rate (K_ON_) from three sensorgrams for analyte concentration ranging from 5–10 μM using the curve-fitting BIAevaluation software, version 3.0 (BIAcore AB) and the 1:1 Langmuir model.

### Luciferase reporter assay

Effect of MAbs on Notch1 signaling was investigated using a functional promoter-reporter assay as described previously^17^,^21^. The cells (HEK293/N1 or N2 MDA-MB-231) were transfected with 790 ng of 12xCSL-Luc and 10 ng pGL3 Basic or 800 ng pGL3 control vector along with 1 ng pRL-Tk (Promega) using Lipofectamine 2000 (Invitrogen, USA). The CCRF-CEM cells were transfected using Turbofect plus (Fermentas, USA) as per the manufacturer’s instructions. Notch signaling in HEK-293hN1/N2 cells was activated by providing pre-coated Jagged1Fc as described previously^17^,^21^. Luciferase reporter activity was estimated 30–36 hours post-transfections using the Dual Luciferase assay kit following the manufactures protocol (Promega, Madison, WI) and a TD-20 Luminometer (Turner Design, Sunnyvale, USA).

### Collection and processing of primary T-ALL samples

Blood samples form the T-ALL patients were obtained from the Kidwai Memorial Hospital, Bangalore, India after obtaining the appropriate clearance from the Institutional Review Board of the Kidwai Memorial Hospital and the human ethics committee of the Indian Institute of Science, Bangalore. The informed consent was obtained from all the subjects. All the experiments were performed in accordance with the relevant guidelines and regulations with prior approval. The peripheral blood lymphocytes were isolated and cultured in RPMI containing 20% FBS at 37 °C for 2–3 days after which the cells were cultured with or without Notch1 MAbs for 72 hours. The proliferation rate was determined by incubating cells with BrdU for 12 hours and determining its incorporation into DNA. The number of CD34^Hi^/CD45^Hi^ sub-population in the cultured cells was determined by Flow cytometry. The genomic DNA from each human sample was used to identify the mutations in the HD and PEST domains of Notch1^19^.

### Cancer sphere assay

MCF-7, MDA-MB-231 and HCT-116 cells (5 × 10^4^ cells/well) were seeded in serum free DMEM-F12 medium with growth factors in a semi-solid medium containing methyl cellulose as described earlier^28^. Effect of MAbs on the sphere forming capacity of these cells was assessed as described previously^17^.

### Cell proliferation assay

Effect of MAbs on cell proliferation was investigated using BrdU incorporation assay. Briefly, 5 × 10^3^ cells were seeded in 96-well plates (Nunc) and incubated in the presence of control IgG or MAbs for 72 hours. The cells were then labeled with BrdU for 12 hours and its incorporation was determined as per the manufacturer’s recommendation (Calbiochem, USA).

### Matrigel invasion assay

*In vitro* cancer cell invasion assay was performed using the BioCoat Matrigel Invasion Chambers as per the manufacturer’s instructions (Becton Dickinson). MDA-MB-231 cells (5 × 10^4^) were grown in the presence of control IgG or MAb604.107 for 72  hours after which they were trypsinized and a total of 50,000 cells were seeded for the invasion experiment for 24 hours. The number of cells invaded were counted and plotted as % cell invasion.

### *In vivo* tumor formation assay

All the *in vivo* experiments were performed in accordance with relevant guidelines and regulations with prior approval from the Animal Ethics Committee (IISc, Bangalore). MDA-MB-231, HCC-1806, BT-474, and HCT-116 cells were injected subcutaneously into female nude mice (1 × 10^6^ cell/mouse) and tumor formation was monitored. The mice were randomly distributed into different groups of four animals once the tumors reached appropriate volumes (100/200/500 mm^3^) and then administered either the control IgG or the MAb (15 mg/kg body weight every 48 hours for 15 to 20 days) intraperitoneally and tumor growth was monitored.

### Statistical analyses

The statistical analyses were performed with the Student t test using graph-pad prism-5 software. The P value of < 0.05 was considered statistically significant.

## Results

### Molecular modeling of T-ALL associated mutant Notch1

**Figure 1:**
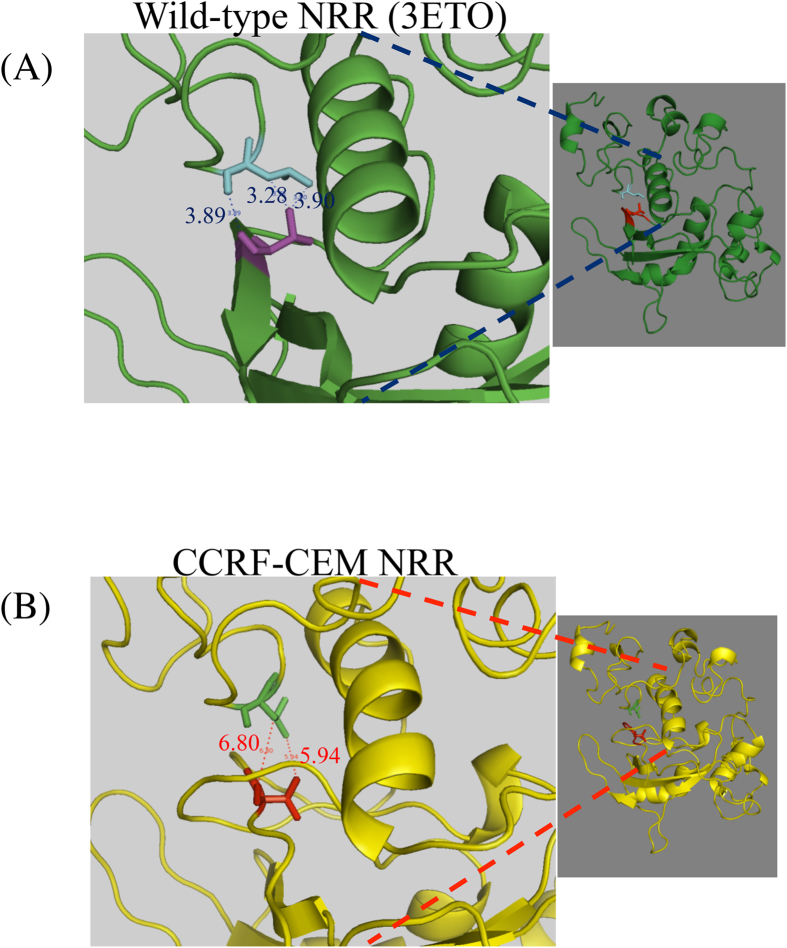
Molecular modeling of Notch1 NRR 3D structural models of the wild-type (3ETO) and mutant (CCRF-CEM) NRR. The interface residues determined were then compared to those in the wild type structure.

Aberrant Notch signaling has been associated with various solid tumors and hematological malignancies with nearly 50% of T-ALL patients harboring the oncogenic “gain-of-function” mutations in Notch1^19^. Effects of these mutations on the conformation of Notch1 NRR was modeled using the mutant Notch1 harboring a 12 amino acid insertion identified in the CCRF-CEM leukemia cell line^19^. As shown in Fig. 1A, in the wild type NRR (closed conformation) the ‘S2-cleavage site’ (V1722) interacted with L1482 of LNR-A. Insertion of 12 amino acids in the HD domain led to opening of the NRR (Fig. 1B), which disrupted the interactions between the HD (V1722) and LNR-A (L1482) paving the way for cleavage at S2-site and ligand-independent receptor activation. The domain-domain interactions data also suggested that insertion of 12 amino acids in the HD affects HD-LNR-A interactions, but not the HD-LNR-B or HD-LNR-C interactions. These results are in agreement with the previous biochemical data^22^ highlighting the implications of these mutations in the Notch structure-function relationship and T-ALL pathogenesis (Fig. 1).

### Anti-Notch1 antibodies can distinguish between the conformations of the mutant and wild-type Notch1 receptors

**Figure 2:**
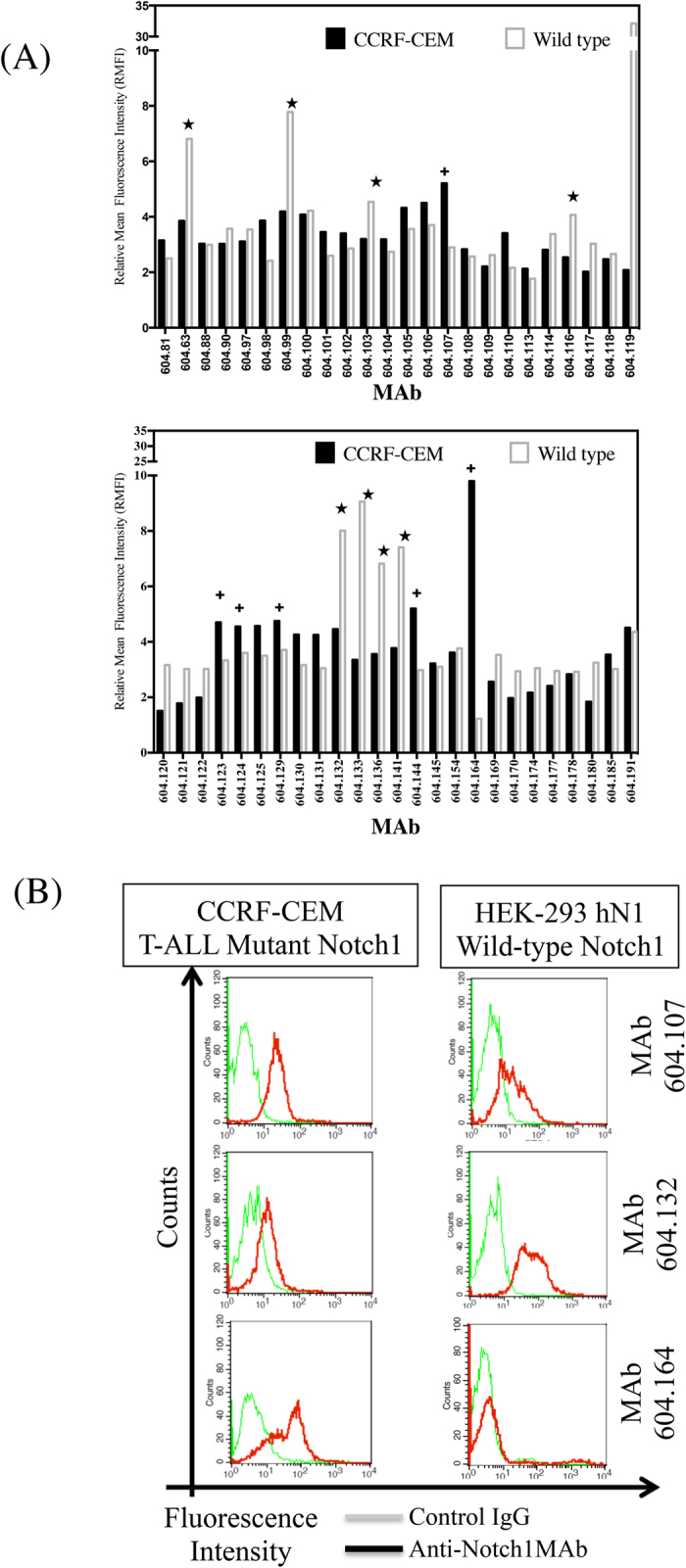
Characterization of Notch 1 NRR specific monoclonal antibodies. (**A**) Notch1 expressing cells CCRF-CEM (Mutant) or HEK-293 Noch1 (wild-type) were incubated with control IgG or anti-NRR-MAbs followed by incubation with anti mouse IgG-FITC conjugate. The MAb binding was determined using the Becton-Dickinson Flow cytometer. (**B**) The HEK293 cells expressing the wild-type Notch1 or CCRF-CEM cells expressing mutant Notch1 were incubated with anti-NRR MAbs 604.107, 604.132 and 604.164, and binding was determined as described Fig. 2A. The Relative mean fluorescent intensity (RMFI) was calculated after normalizing with the control IgG; results are expressed as Means ± S.D.; n = 3.

Forty-nine MAbs raised against wild type Notch1 NRR were further characterized for their ability to recognize either the wild type Notch1 (HEK293-hN1) or the mutant Notch1 (CCRF-CEM) in flow cytometry-based assays. As shown in (Fig. 2A), several MAbs marked* preferentially recognized the wild-type receptor while five MAbs marked** + **were mutant-specific. Several MAbs exhibited similar binding to both wild type and mutant receptor conformations. Identification of MAbs exhibiting differential binding to the mutant and wild-type Notch1 further confirmed existence of mutation-induced conformational changes in the NRR as predicted by the model. The mutant specific MAbs (604.107 and 604.164) and the wild-type specific MAb (604.132) were chosen for functional characterization. As shown in Fig. 2B, MAb 604.107 exhibited relatively higher binding to T-ALL cell line, but could also bind to the wild type receptor also. The MAb 604.164 could bind to the T-ALL cells, but showed no binding to the wild type Notch1. In contrast, the MAb 604.132 recognized the wild type, but did not recognize the mutant Notch1. As shown in Table 1, the *K*_*D*_ of MAb 107 for the wild type receptor (5.08 ± 1.09 × 10^−8^ M) was ten fold higher than that for the mutant (L1594P) receptor (6.41 ± 0.12 × 10^−9^ M). Similarly, the K_D_ of MAb 164 was 100 fold higher for the wild type (2.03 ± 0.48 × 10^−7^ M) than for the mutant (1.93 ± 0.12 × 10^−9^ M). In contrast, the K_D_ of MAb 132 was 10 fold higher for the mutant (4.02 ± 1.12 × 10^−8^ M) compared to the wild type (4.95 ± 0.84 × 10^−9^ M). These data are in agreement with the original characterization of the antibodies and clearly indicate that these MAbs can differentiate conformations of different mutants of Notch1 NRR.

### Effect of anti-Notch1 NRR antibodies on the wild-type and mutant Notch1 signaling

**Figure 3:**
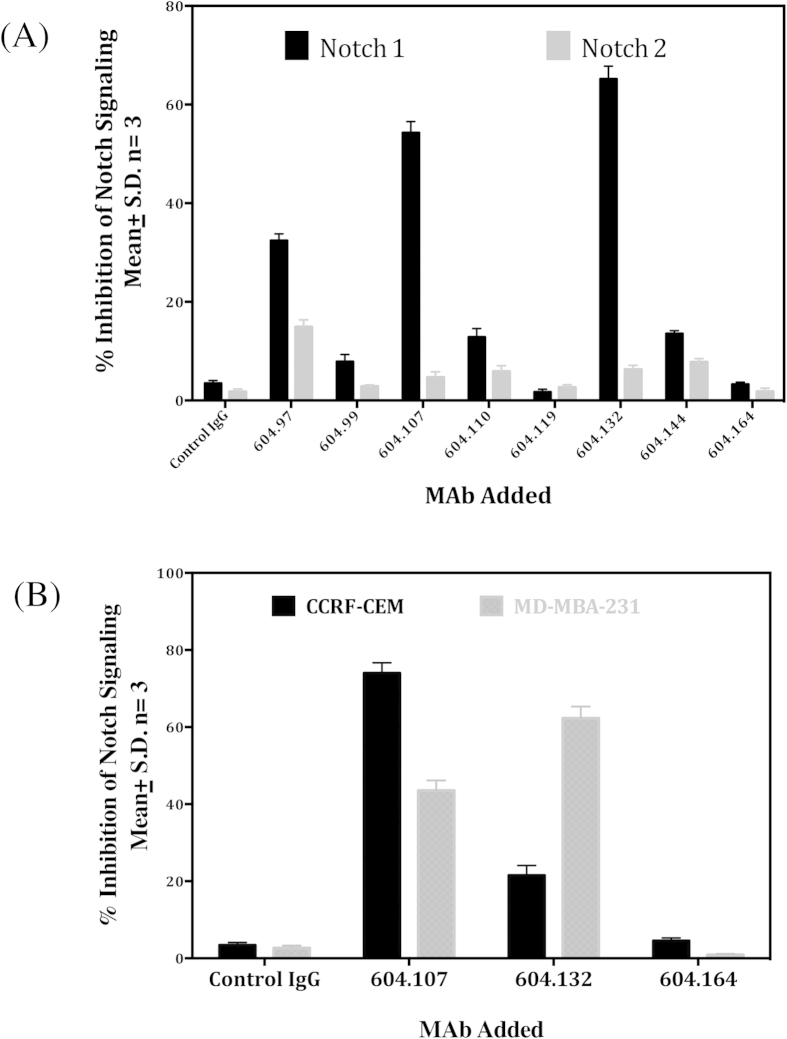
Effect of MAbs on Notch signaling. (**A**) Stable cell lines expressing Notch1 and Notch 2 were transfected with 12xCSL-Luc in the presence of Jagged1-Fc pre-coated on the plastic surface and incubated with the control or Anti- NRR MAbs (IgG 10 μg/ml) and the reporter activities were determined after 48 hours. (**B**) MDA-MB-231 and CCRF-CEM cells were transfected with 12xCSL-Luc reporter plasmid and incubated with the control IgG or MAb (10 μg/ml) and the reporter activities were determined after 48 hours; results are expressed as Means ± S.D.; n = 3.

The Notch1 and Notch2 expressing HEK-293 cells were incubated with Jagged1-Fc that was adsorbed on plates and Notch activation was determined in the presence of various Notch1 MAbs. The MAbs 604.107 and 604.132 inhibited ligand-dependent Notch1 signaling, but had no effect on Notch2 activation (Fig. 3 top panel) demonstrating the specificity of the antibodies. Since Notch1 functions as an oncogene in both solid tumors, as well as, hematological malignancies^5^,^6^, effects of MAbs on solid tumors and hematological malignancies were investigated using different cell lines. At 10 μg/ml, the MAbs 604.107 caused 76% and 44% inhibition of the basal Notch1 signaling in the mutant (CCRF-CEM) and the wild type (MDM-MB-231) Notch1 expressing cells respectively while the MAb 604.132 showed 22% and 64% inhibition respectively. In contrast, MAb 604.164 did not cause any inhibition of Notch signaling with both cell lines (Fig. 3 bottom panel)

**Figure 4:**
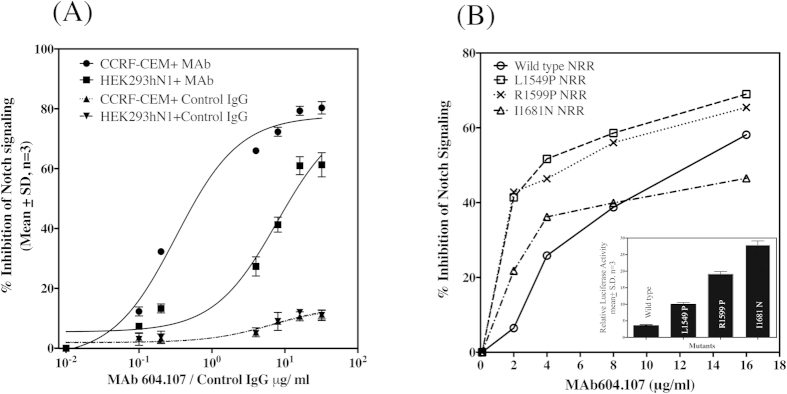
Effect of MAb604.107 on the mutant Notch1 signaling. (**A**) Notch1 expressing cells CCRF-CEM (Mutant) or HEK-293 Noch1 (Wild Type) were incubated with increasing concentrations of control IgG or MAb 604.107. The effect of antibody on Notch1 singling was determined in luciferase assay as described in Fig. 3A. The IC_50_ of MAb for CCRF-CEM was significantly different from IC_50_ for HEK203hN1 (Mean ± S.E 0.32 ± .07 vs 7.8 ± 0.11 μg/ml respectively, p < 0.01). (**B**) HEK 293 cells were co-transfected with 12xCSL-Luc reporter plasmid and the wild-type or mutant (L1594P, R1599P and I1681N) Notch1 cDNAs and then incubated with increasing concentrations of the MAb604.107 and the basal reporter activities were determined after 48 hours. The inset shows the relative Notch signaling of different activating mutations in the Notch 1 NRR; results, means ± SD; n = 3.

Since the MAb 604.107 inhibited Notch signaling in both hematological and solid cancer cell lines, it was chosen for further characterization. When dose dependent effect of the MAb on Notch signaling was investigated, the CCRF-CEM cell line was more sensitive to the antibody than MDA-MB-231 with IC_50_ for the former being nearly 24 times lower than that for the latter (0.32 μg/ and 7.85 μg/ml respectively) (Fig. 4A). Thus, this MAb is ideal for inhibiting the “gain of function” mutants associated with the T-ALL^22^. As shown in Fig. 4B **(inset)**, the HEK 293 cells expressing the full-length Notch1 harboring L1594P, R1599P and I1681N mutations had 3, 6, and 9-fold increase respectively in the basal Notch signaling compared to the wild type. As shown in the Fig. 4B, these mutants were more sensitive to the MAb with marked decrease in the Notch signaling at very low concentrations of the antibody. As the saturating concentrations, the MAb was effective in inhibiting signals in all mutants as well as the wild type.

**Figure 5:**
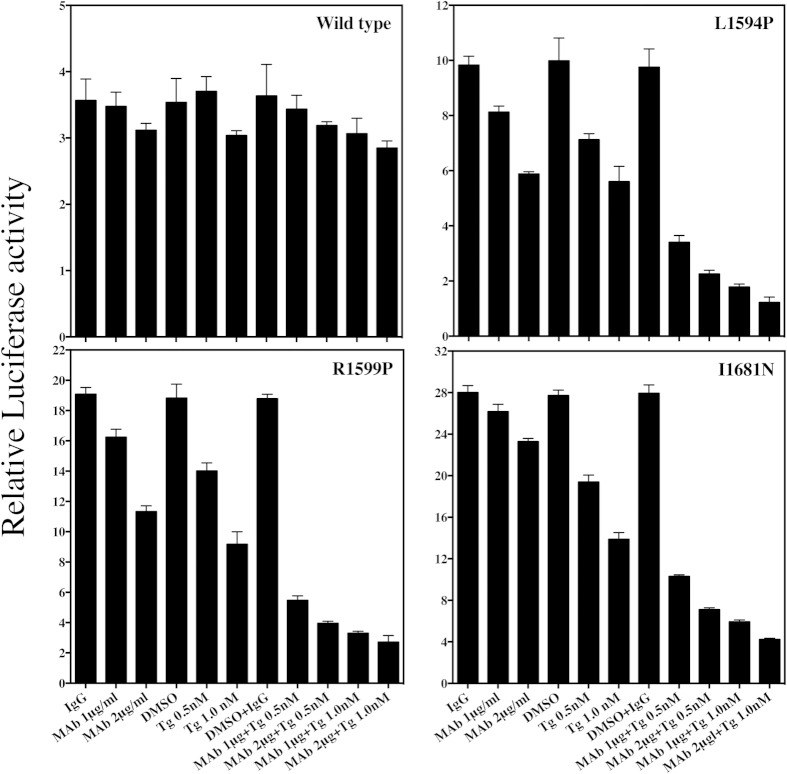
Combinatorial effect of on MAb604.107 and Thapsigargin on the Wild Type and the mutant Notch1 receptors. HEK 293 cells were co-transfected with 12xCSL-Luc reporter plasmid and the Wild Type (**A**) L1594P (**B**) R1599P (**C**) or I1681N (**D**) Notch1 cDNAs followed by incubation with 0.5 or 1 nM SERCA inhibitor Thapsigargin and 1 or 2 μg/ml of MAb604.107 alone or in various combinations and the reporter activities were determined; results expressed as Means ± S.D.; n = 3. In all cases, the combination of Thapsigargin and MAb 604.107 was significantly more effective in inhibiting Notch signaling than the individual reagent (p < 0.001).

Recently, SERCA (Sarco/endoplasmic reticulum Ca^2+^-ATPase) inhibitor Thapsigargin has been reported to impair mutant Notch1 signaling by down-regulating the cell-surface expression of mutant Notch1^29^. Whether the MAb potentiates the effect of Thapsigargin was next investigated. As shown in Fig. 5, combination of Thapsigargin and MAb604.107 significantly augmented the inhibition of the mutant Notch1 (L1594P, R1599P and I1681N) signaling compared to the inhibition caused by either the MAb or Thapsigargin alone. However, at these same concentrations, combinatorial treatment had marginal effect on the wild-type Notch1 signaling. These data clearly demonstrated the effectiveness of the MAb604.107 as a specific inhibitor of T-ALL associated “Gain Of Function” Notch1 mutations.

### Effect of MAB 604.107 on proliferation and Leukemia Initiating Cell (LIC) sub-population of T-ALLs

**Figure 6:**
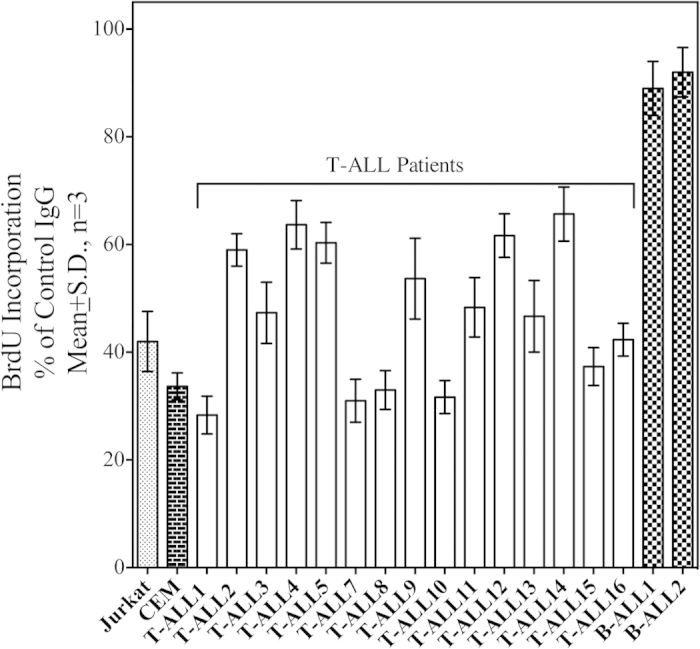
Effect of anti-Notch1 antibodies on the primary T-ALL Cells. The primary T-ALL or B-ALL cells obtained from patients as well as the Jurkat and CCRF-CEM cell lines were incubated with control IgG or the MAb 604.107 (10 μg/ml) for 72 hours and BrdU incorporation into DNA was determined. The incorporation obtained in presence of MAb for each sample was compared to that obtained with the control IgG for the same sample which was considered as 100%; results expressed as Means ± S.D.; n = 3. The MAb significantly inhibited proliferation of cells in case of all human TALL samples (p < 0.01).

As discussed above, the Gain-of-function mutations in Notch1 leading to ligand-independent constitutive activation of the receptor play an important role in the oncogenesis of a large number of T-ALLs^19^. In the present study, 5 out of 14 T-ALL patients harbored single point non-synonymous mutations in the HD domain while one patient exhibited a truncating mutation in the PEST domain (Supplementary Figure 1). The PBLs obtained from each patient were cultured in presence of the control IgG or the MAb and the rate of proliferation was determined using BrdU incorporation. As shown in the Fig. 6, the MAb (10 μg/ml) inhibited proliferation of Jurkat and CCRF-CEM cell lines and also that of all T-ALL patient-derived cells, but had absolutely no effect on the primary B-ALL cells suggesting the effectiveness of MAb in inhibiting the gain of function mutations.

**Figure 7:**
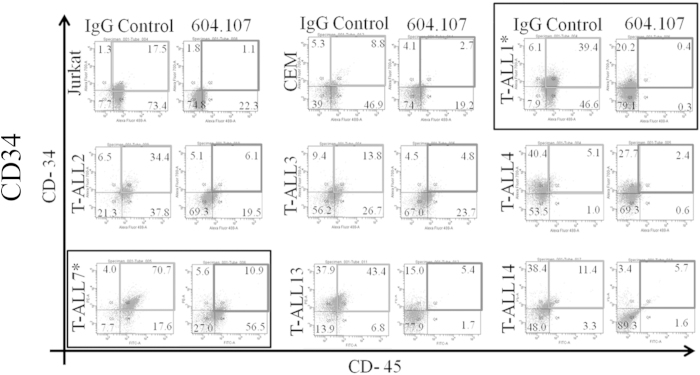
Effect of anti-Notch1 antibodies on the Leukemia Initiating cells. The Jurkat and CCRF CEM cell lines, as well as the Peripheral blood lymphocytes isolated from the seven different T-ALL patients were cultured in the presence of the control IgG or MAb604.107 (10 μg/ml) for 72 hours. The proportion of the cells expressing of the cell surface markers CD34 and CD45 was determined by flow cytometry using the specific antibodies; results, means ± SD; n = 3. The paired T analysis of all human cells revealed significant decrease in CD34/CD45 cells upon incubation with MAb (p = 0.0188).

The CD34^High^/CD45^High^ sub-population constitute the Leukemia Initiating cell (LIC) in T-ALL^30^. Effect of MAb604.107 on LICs from the patient- derived primary T-ALL cells, as well as, the mutant Notch1 bearing cell line (CCRF-CEM) was next investigated. The cells were incubated with the MAb (10 μg/ml) for 48 hours and the fraction of CD34^High^/CD45^High^ cells was determined by flow cytometry. As shown in Fig. 7, the patient-derived T-ALL samples exhibited significant variability in the fraction of CD34^High^/CD45^High^ cells ranging from 5% to 70%. However, the MAb treatment significantly depleted this population in each of the 7 patient-derived primary T-ALL samples as well as in the cell lines tested. In another experiment, the CD34^High^/CD45^High^ cells sorted out from the patients’ PBL showed much higher inhibition of proliferation compared to the rest of the cells clearly indicating the effectiveness of the MAb on LIC subpopulations (Supplementary Figure 2A). Moreover, MAb604.107 also inhibited the Notch targets genes (*Hes1 and NRARP*) in CCRF-CEM cells (Supplementary Figure 2B).

The above data indicate that the MAb 604.107 specifically inhibits Notch signaling in the ‘gain of function’ mutants associated with T-ALL cases and has a therapeutic potential for treating this hematological malignancy.

### Effect of MAb604.107 on solid cancers: proliferation, EMT and CSCs in solid tumors

**Figure 8:**
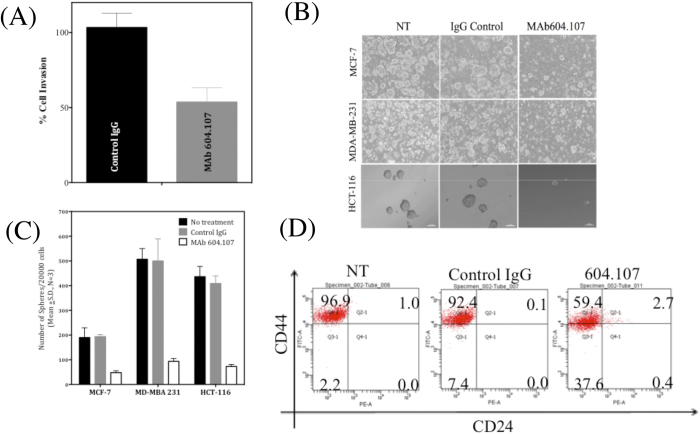
Effect of anti-NRR antibodies on the breast cancer cells. (**A**) MDA-MB-231 cells were pre-treated with the control IgG or MAb604.107 (20 μg/ml) and then seeded in Matrigel Invasion Chambers. The total cells were counted after 24 hours to determine the % invasion. (**B**) MCF-7, MDA-MB-231 and HCT-116 cells (5 × 10^4^) were cultured in semi-solid medium (methyl cellulose) in the presence of the control IgG or MAb604.107 (20 μg/ml) and the number of mammospheres formed was determined; magnification 10x. (**C**) Quantification of the sphere forming capacity of MCF-7, MDA-MB-231 and HCT-116 cells in the presence or absence of the MAb. (**D)** MDA-MB-231 cells were treated with the control IgG or MAb (20 μg/ml) for 72 hours and expression of the cell surface markers CD44 and CD24 was analyzed by flow cytometry using the specific antibodies; results expressed as Means ± S.D.; n = 3.

The Notch signaling pathway is aberrantly activated in several solid tumors^31^,^32^. Since the MAb 604.107 also interacted with the wild type Notch, although with relatively lower affinity, it was interesting to find out effect of this MAb on different solid cancer cell lines. First, effect of the MAb on proliferation of the breast cancer cell line was investigated. Incubation with MAb (20 μg/ml) for 72 hours resulted in approximately 70% decrease in BrdU incorporation (data not shown) and 45% decrease in the invasive potential of MDA-MB-231 cells (Fig. 8A). Since the Notch signaling plays an important role in the self-renewal of normal and cancer stem cells of epithelial origin^33^,^34^, effect of MAb 604.107 on the putative breast and colon cancer stem cells (CSCs) was determined using an *in vitro* sphere formation assay. The breast cancer cells MCF-7 and MDA-MB-231, and the colon cancer cells HCT-116, were cultured in the presence of control IgG or MAb (10 μg/ml) in methylcellulose medium and the number of spheres formed was counted. MAb604.107 significantly inhibited the sphere formation in three cancer cell lines (Fig. 8B,C). In concordance with our previous study^17^, pre-treatment with MAb604.107 irreversibly affected the secondary spheres formation even after antibody removal. This confirms the effect of MAb on CSC subpopulation in these cells (Supplementary Figure 3). The MAb also depleted the CD44^Hi^/CD24^Low^ cells^35^, the putative Cancer Stem cells in the MDA-MB-231 cells (Fig. 8D). The antibody treatment decreased levels of the cleaved-Notch1 and the downstream effector of Notch signaling Hes-1 in both MDA-MB-231 and HCT 116. Finally, the MAb increased levels of Caspase 3, while decreasing the expression of anti-apoptotic marker, BCl2 in both cell lines (Supplementary Figure 4). These data clearly show that the MAb increases apoptosis of the cancer cell lines by inhibiting the Notch1 signaling and also targets the breast CSCs.

### Effect of anti-Notch1 MAb on the chemo-resistant Notch1^High^ sub-population of cancer cells

**Figure 9:**
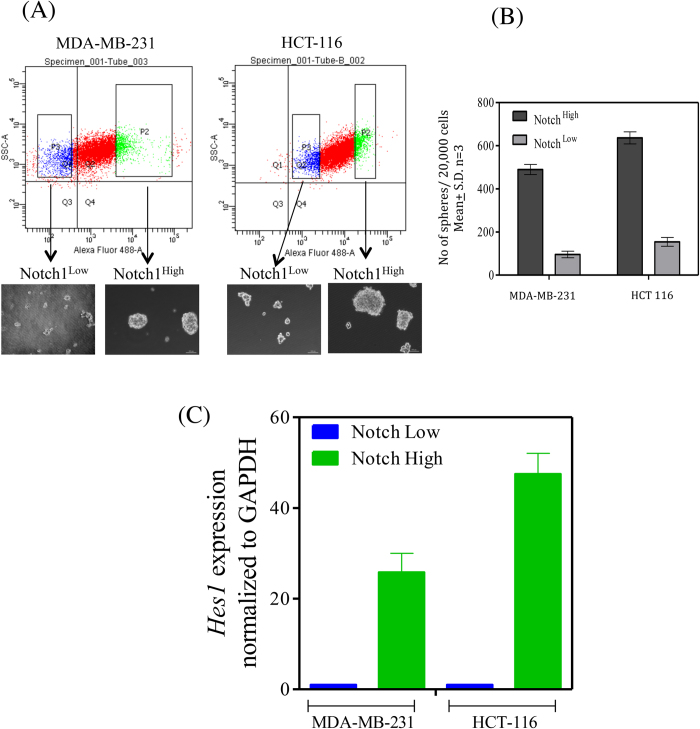
Heterogeneity of Notch1 expression in the breast and colon cancer cells. (**A**) MDA-MB-231 or HCT-116 cells were incubated with polyclonal Notch1 NRR antibody followed by incubation with the anti-rabbit FITC conjugate and sorted on the basis of the fluorescence intensity to obtain Notch^High^ and Notch^Low^ cells from the total cell population. The ‘Flow sorted’ Notch^High^ and Notch^Low^ cells (5 × 10^4^) were cultured in a suspension medium and the sphere forming capacity was determined. (**B**) Quantitation of data shown in Fig. 9A. (**C**) Total RNA was isolated from the ‘Flow-sorted’ Notch^High^ and Notch^Low^ cells and expression of *HES-1* transcript was determined by Q-PCR; results expressed as Means ± S.D.; n = 3.

**Figure 10:**
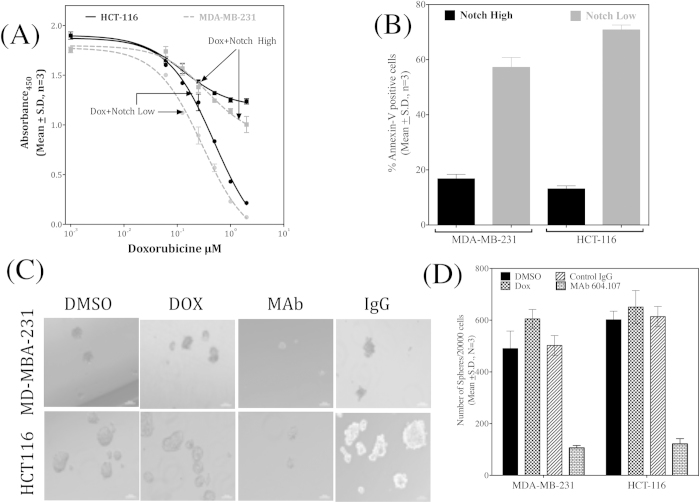
Effect of anti-Notch1 antibody on Notch1^High^ chemo-resistant stem-like cells. (**A**) Notch^High^ and Notch^Low^ cells isolated as described above were cultured in the presence of increasing concentrations of Doxorubucin and BrdU incorporation was determined. (**B**) The Notch^High^ and Notch^Low^ obtained from both cell line were stained with Annexin V-PE-Cy5 and analyzed by flow cytometry for apoptotic cell death. (**C**) Notch^High^ and Notch^Low^ cells (5 × 10^4^) were seeded in the presence of Doxorubucin or MAb604.107 and the sphere formation capacity was determined. Appropriate controls were used. (**D**) Quantitation of data shown in Fig. 10C; results expressed as Means ± S.D.; n = 3.

A strong co-relation exist between Notch1 signaling in the lung CSCs and chemotherapy resistance^36^. A possible role for similar high Notch1 expression in the breast and colon cancer cells was investigated. The MDA-MB-231 and HCT-116 cells were ‘flow-sorted’ on the basis of Notch1 expression and their sphere forming capacity and drug resistance properties were determined. The Notch1^High^ sub-populations from both these cell lines showed enrichment of sphere forming cells (Fig. 9A,B) and exhibited elevated *Hes-1* expression compared to the Notch1^Low^ cells confirming differential Notch activities in these sub-populations (Fig. 9C). The Notch1^High^ cells also exhibited higher resistance to Doxorubucin treatment whereas the Notch1^Low^ sub-population remained sensitive to the drug (Fig. 10A). In addition, Doxorubucin treatment led to significantly higher Annexin-V positive cells in Notch1^Low^ sub-population compared to Notch1^High^ cells (Fig. 10B) reiterating the fact that Notch1^High^ cells are chemotherapy resistant in these solid tumors as well. Interestingly, treatment with MAb604.107 reduced the sphere forming capacity of the chemotherapy-resistant Notch1^High^ sub-population (Fig. 10C). Further, EMT and stemness genes were up-regulated in Notch1^High^ sub-population (supplementary Figures 5 & 6), suggesting the clear involvement of Notch1 signaling in EMT and CSCs in breast and colon cancer cells. Further, Doxorubucin resistant cells were established culturing the both MB-MDA-231 and HCT116 cell lines in presence of Doxorubucin (10 μM) for three weeks. The Doxorubucin resistant cells exhibited higher Notch activity (Supplementary figure 7). While the Doxorubucin sensitive cells remained sensitive to both MAb and the drug, the drug had no effect on the resistant cells, but the MAb had a marked effect on the proliferation of the Doxorubucin resistant cells (Supplementary figure 7). These results revealed the existence of heterogeneity in terms of Notch1 expression in the breast and colon cancer cells and the importance of targeting Notch1 signaling to overcome drug-resistance in these cancers.

### MAb604.107 augments the efficacy of chemotherapy treatmen**t**

**Figure 11:**
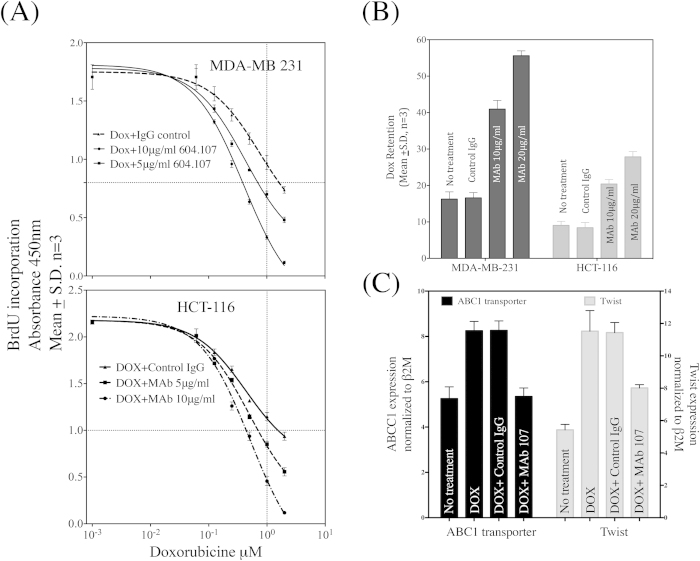
MAb604.107 improves the efficacy of chemotherapeutic treatment. (**A**) MDA-MB-231 and HCT-116 cells (1 × 10^4^) were cultured with increasing concentrations of Doxorubucin in the presence and absence of MAb604.107 (5 or 10 μg/ml). BrdU incorporation was determined after 72 hours of incubation. (**B**) MDA-MB-231 and HCT-116 cells (1 × 10^5^/well) were incubated with the control IgG or MAb (10 or 20 μg/ml) and Doxorubucin was added after 24 hours. Post 36 hours incubation; Doxorubucin incorporation was determined using the flow cytometric assay. (**C**) MDA-MB-231 cells were incubated with Doxorubucin for 24 hours in the presence of the control IgG or MAb604.107. RNA was isolated and expression of (**D**) ABCC1 and (**E**) Twist was determined by Q-PCR; results expressed as Means ± S.D.; n = 3. The difference between the effects of the control IgG and MAb 604.107 was significantly different (p < 0.01).

Since Notch1^High^ cells exhibited chemotherapy resistance, but were responsive to inhibition of the Notch signaling by the MAb, effect of combination of the drug and MAb on the breast and colon cancer cell lines was next investigated. The cells were cultured with increasing concentration of the drug in presence and absence of MAb (5 or 10 μg/ml) for 72 hours and rate of proliferation of the cells was determined by BrdU incorporation. As shown in Fig. 11A, the MAb significantly increased the effectiveness of the drug. The antibody also increased the sensitivity of both cell lines to Doxorubucin with nearly 4-fold decrease in the IC_50_ for the drug.

To determine the mechanism of increase in the sensitivity to the drug, both cells were pre-incubated with the control IgG or MAb for 24 hours followed by incubation with Doxorubucin for 12 hours, at the end of which the intracellular retention of the drug was determined by flow-cytometry. As shown in Fig. 11B, MAb treatment significantly enhanced the intracellular retention of the drug. It is also known that Notch1 and Twist1 up-regulate expression of the multidrug resistance gene ABCC1 in the breast cancer cells^37^,^38^. As shown in the Fig. 11C, there was enhanced expression of ABC1 and Twist in presence of Doxorubucin, which was decreased in the presence of the MAb. Thus, inhibition of Notch1 signaling decreased ABCC1 levels resulting decreased efflux of the drug leading to increased sensitivity of the cells to the Doxorubucin.

### Effect of MAb604.107 treatment on tumor growth *in vivo*

**Figure 12:**
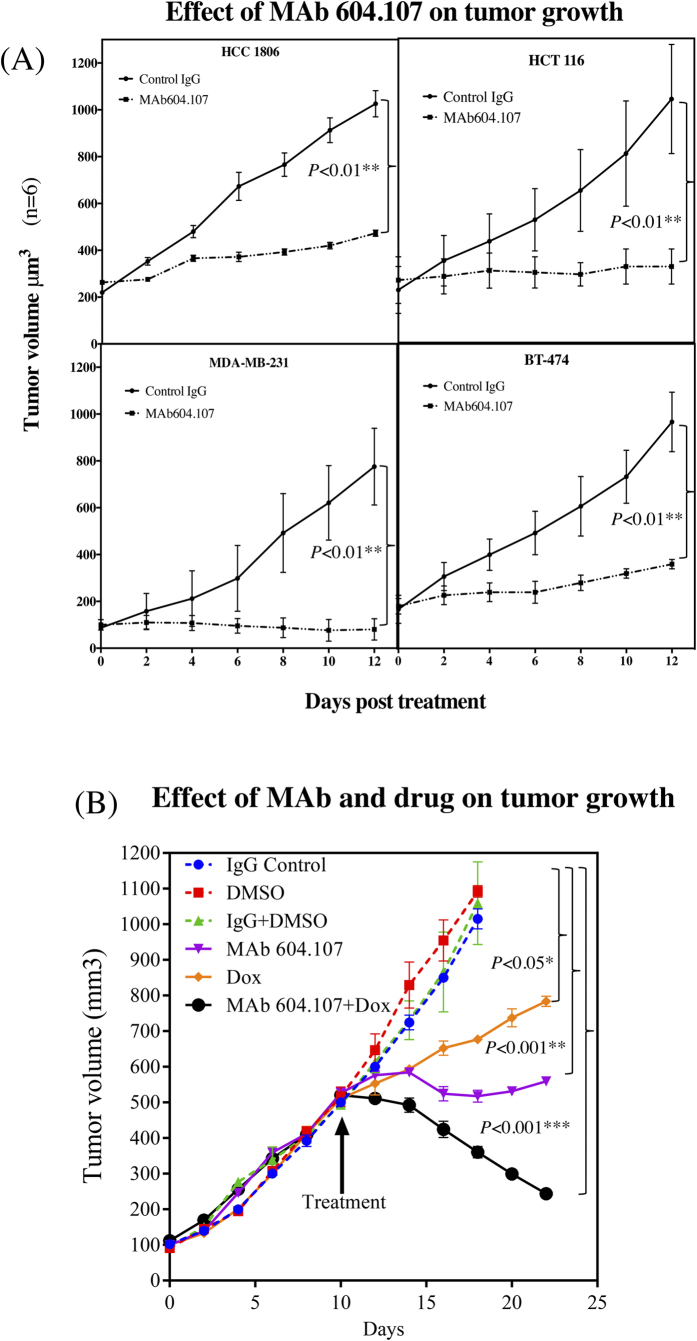
*In vivo* effects of anti-Notch1 antibodies on the tumor growth. (**A**) MDA-MB-231, HCC-1806, BT-474 and HCT-116 (1 × 10^6^) were injected into nude mice and allowed to attain volume of 100–200 mm^3^ The Control IgG or MAb was administered to the animals (15 mg/kg body weight.) every 48 hours for 12 days and the tumor size was determined. (**B**) HCT-116 cells were injected into the nude mice and the tumors were allowed to attain the volume of 500 mm^3^. The animals were then intra-peritoneally treated with Doxorubucin (3 mg/kg body weight), MAb (15 mg/kg body weight) alone or in combination with the appropriate controls and tumor size was determined; results expressed as Means ± S.D.; n = 6. The p values are shown in the figure.

The effect of MAb604.107 treatment on tumor growth *in vivo* was examined using four different cell lines. MDA-MB-231, HCC-1806, BT-474 and HCT-116 tumor xenografts were first allowed to develop to approximately 200 mm^3^ followed by intraperitoneal injection of either the control IgG or MAb (15 mg/kg body weight). As shown in Fig. 12, the MAb treatment inhibited further tumor growth all four xenografts. In addition, the MAb treatment significantly inhibited the genes associated with the Notch pathway and stemness (Supplementary figure 8) and cleaved Notch1 expression in the resected tumor xenografts (Supplementary figure 9).

Effect of combinatorial treatment of MAb and Doxorubucin was next evaluated in HCT-116 xenografts. The xenografts were allowed to grow to approximately 500 mm^3^ and then administered intraperitoneally Doxorubucin (3 mg/kg body weight) and the control IgG or MAb604.107 (15 mg/kg body weight) alone or in combination, every 48 hours. As shown in Fig. 12B, Doxorubucin treatment led to a reduced tumor growth while the MAb inhibited tumor growth completely. Interestingly, the combination of MAb and Doxorubucin led to a significant regression of the pre-formed tumor in these mice, clearly indicating the effectiveness of the combinatorial therapy.

## Discussion

Notch signaling plays an important role in carcinogenesis and is implicated in CSC maintenance^7^,^39^,^40^. While in case of solid cancer, overexpression of Notch has been thought to be the causative factor; the gain-of-function mutations in Notch1 NRR have been shown to be the responsible factor in case of hematological malignancies. Notch1 antibodies, by their ability to target CSCs^9^,^17^, are ideal therapeutic tools. In the present study, we report a MAb with therapeutic potential for both T-ALL, as well as, the breast and colon cancers.

The “Gain-of-function” mutations in Notch1 NRR have been shown to be responsible for hematological malignancy. As shown in the present study, 5 out of 14 T-ALL patients investigated showed mutations that have been already reported and as shown in Fig. 4B, these mutations caused increase in the basal, ligand independent Notch activity ranging from 3 to 9 fold higher than the basal activities with I1681N being the most severe mutation causing highest increase in the activity. The modeling of mutant Notch NRR present in the CCRF-CEM cell line shows that insertion of 12 amino acids in NRR results in opening of the NRR exposing the cleavage sites in the NRR making it more susceptible for S2 cleavage, thus accounting for the higher basal Notch activities. As shown in this study, the MAbs that preferentially target such conformations are ideal for cancer immunotherapy.

Of the several NRR MAbs generated in this study, two MAbs interacted with CCRF-CEM NRR with much higher affinities. One of these MAbs (604.164) exhibited 100-fold higher affinity for the mutant NRR compared to that for the wild type NRR with virtually no binding to the wild type receptor. However, this MAb had absolutely no effect on the mutant Notch signalling suggesting the possibility that the epitope recognized by the MAb may not participate in Notch signaling. This ruled out its usage as a potential therapeutic antibody. Further, we employed phage-display screening and ELISA to determine the putative epitope of these antibodies. As shown in supplementary figure 10 and supplementary table-1 inhibitory MAb 604.107 binds to LNR-A and HD domain while MAb 604.164 binds only to HD domain of Notch1 NRR. It is interesting to note that the previously described inhibitory anti-Notch1 antibodies^18^ also co-crystalize with LNR-A and HD domain. Therefore, it is tempting to speculate that inhibitory antibodies lock the receptor in an auto-inhibited conformation by interacting with LNR-A and HD domains.

In contrast to MAb 604.164, MAb 604.107 recognized both mutant and wild-type Notch1 with 10 fold higher affinity for the mutant receptor and inhibited the ligand independent signaling of both mutant and wild type Notch, with IC_50_ for the mutant Notch being nearly 14 times lower than for the wild type. Thus, at lower concentrations, the antibody was effective in inhibiting mutant signaling without having any effect on the wild type Notch1. This was confirmed using the T-ALL Notch1 mutants. As shown in the Figs 4,5, different mutations caused different degrees of increase in the basal Notch1 signaling. The mutants were relatively more sensitive to the MAb. However, the mutants were much more sensitive to low concentrations of the antibody in presence of Thapsigargin that has been recently identified as preferential inhibitor of Notch1 receptors with mutated NRRs. The MAb can increase effectiveness of Thapsigargin and make the cells with extremely sensitive to the drug at the concentrations of both at which the wild type receptor are not affected at all.

The MAb also inhibited proliferation of Jurkat and CCRF-CEM cell lines and all PBLs derived from T-ALL patients, but had no effect on the B-ALL cases. The MAb604.107 depleted LIC (CD34^High^/CD45^High^) subpopulation in the T-ALL patient derived primary cells, as well as in the cell lines. This observation clearly suggests the importance of oncogenic Notch signaling in maintenance of the LIC. All these data clearly show the therapeutic potential of the MAb specifically targeting of T-ALL associated mutant Notch1 signaling.

The putative epitope of MAb604.107 appears to reside in LNR-A and HD domains (data not shown). What is very clear from these data is that there are few “drugable” epitopes in NRR that need to be targeted. The epitope of MAb 107 is clearly the interesting epitope while that of MAb 164 is not.

MAb 604.107 can also be useful in treating solid cancers and as discussed above there is clearly an effect on the wild type Notch signaling although requiring higher concentrations of the antibody. It is known that the metastatic tumors are highly differentiated and remain refractory to chemotherapeutic agents^8^,^41^. Notch-dependent drug resistant CSCs sub-population has been demonstrated in various cancers^42^,^43^. Of the several strategies targeting Notch signaling in CSCs^44^ targeting of the paralogous Notch receptor using Notch antibodies is ideal for therapeutic interventions^9^,^17^. Here we demonstrate that Sphere formation, which is a characteristic feature of the putative CSC sub-population in the breast and colon cancers^45^,^46^, was effectively inhibited by the MAb604.107. The MAb also depleted the chemotherapy and radiotherapy resistant CD44^High^/CD24^Low^ subpopulation in the MDA-MB-231 cells^47^ while down regulating expression of genes associated with stemness and EMT. In addition, the MAb inhibited invasiveness of MDA-MB-231 and migration through Matirgel suggesting a potential therapeutic application of anti-Notch1 antibodies in inhibiting the process of EMT in aggressive solid tumors.

Despite extensive studies on the role of Notch1 signaling in CSC and drug resistance, association of Notch1 activity with CSC phenotype remains elusive. Recently, Hassan *et al.*, correlated Notch1 activity with the cancer stem-like properties and poor survival rate in lung adenocarcinoma^36^. Notch1^High^ sub-population in breast and colon cancers also demonstrate enhanced sphere forming efficiency and higher level of genes associated with stemness and EMT. Our results suggest the de-differentiation in Notch1^High^ sub-population into Notch1^Low^ cells and vice versa post one-week culture (Sharma and Dighe Unpublished). Therefore, it will be interesting to investigate the implication of de-differentiation in tumorigenesis and therapeutic resistance. Interestingly, Notch1^High^ sub-population remains refractory to Doxorubucin treatment, both in terms of proliferation and apoptotic cell death, but exhibit sensitivity to MAb treatment. These results indicated that MAb might improve the efficiency of chemotherapeutic drugs. As shown in Fig. 11, the MAb604.107 augmented the efficacy of Doxorubucin treatment leading to decrease in IC_50_ value of the drug four fold. MAb604.107 treatment also resulted in significant intracellular retention of Doxorubucin by down-regulation of the Notch dependent up-regulation of ABCC1 transporter and Twist expression and consequent reduction of drug efflux making the cells sensitive to the drug.

Tumor initiating cells (TIC) are the hallmark of *in vivo* tumor growth^41^. Pre-treatment of MDA-MB-231 and HCT-116 cells with MAb604.107 completely inhibited tumor growth *in vivo* suggesting that MAb depleted TIC sub-population in these cells (data not shown). Further, MAb604.107 also impeded *in vivo* tumor growth of xenografts of MDA-MB-231, BT-474, HCC-1806 and HCT-116 cells. Further, combinatorial treatment of MAb and Doxorubucin of HCT-116 xenografts resulted in significant regression of the pre-formed tumor validating the effectiveness for Notch1 MAb in adjuvant chemotherapy. Thus, the MAb characterized in this study not only can effectively block “Gain- of function” mutant Notch1 signaling due to its higher affinity, but also affect the cancer cells with higher expression of Notch1 with inhibition of Notch signaling in addition to making them more sensitive to the drugs by increasing retention of the drugs.

In summary, despite previous attempts on the generation of anti-Notch1 MAbs, the monoclonal antibody reported here has several interesting features (Supplementary Table-2). Most important being it’s ability to preferentially targets mutant Notch1 receptors due to its higher affinity for such receptors. It can make the drugs such as Thapsigargin effective at concentrations that have no effect on the wild type. Although its affinity for the wild type receptors is relatively lower, it does affect the cells that exhibit higher Notch1 expression. It also made the cells sensitive to drugs such as Doxorubucin with decreasing the IC_50_ four fold by increasing the cellular retention drug. The MAb also retarded the growth of pre-formed tumors and in presence of chemotherapeutic drug actually regressed the tumors. Ability of the antibody to reduce expression of ABCC1 transporter perhaps made the tumor sensitive to Doxorubucin. Thus, this study reports an interesting versatile MAb that can be a very effective in different cancers.

## Additional Information

**How to cite this article**: Sharma, A. *et al.* A novel Monoclonal Antibody against Notch1 Targets Leukemia-associated Mutant Notch1 and Depletes Therapy Resistant Cancer Stem Cells in Solid Tumors. *Sci. Rep.* **5**, 11012; doi: 10.1038/srep11012 (2015).

## Supplementary Material

Supplementary Information

## Figures and Tables

**Table 1:** **Affinities of MAbs for mutant and wild-type Notch1 NRR.**

**MAb**	**WT Notch1**	**L1594P**
604.107	5.08 + 1.09 × 10^−8^M	6.41 + 0.12 × 10^−9^ M
604.132	4.95 + 0.84 × 10^−9^M	4.02 + 1.09 × 10^−8^ M
604.164	2.03 + 0.48 × 10^−7^M	1.93 + 0.12 × 10^−9^ M

Wild-type or mutant (L1594P) NRR proteins were coated on CM5 chip and allowed to interact with anti-NRR MAbs. The affinity constants for each MAb were calculated from the sensogram obtained using BIAevaluation3.0.
