# Supplementary material for: A novel Monoclonal Antibody against Notch1 Targets Leukemia-associated Mutant Notch1 and Depletes Therapy Resistant Cancer Stem Cells in Solid Tumors
Source: Sci Rep. 2015 Jun 5;5:11012. doi: 10.1038/srep11012 (PMC4457015; doi:10.1038/srep11012)
Supplement: Supplementary Information [file srep11012-s1.pdf]

Supplementary Information:-

# A novel Monoclonal Antibody against Notch1 Targets Leukemia-associated Mutant Notch1 and Depletes Therapy Resistant Cancer Stem Cells in Solid Tumors

**Ankur Sharma[1], Rupali A Gadkari[2], Satthenapalli V Ramakanth[1$], Krishnanand Padmanabhan[1$], Davanam S Madhumathi[3], Lakshmi Devi[3], Lingappa Appaji[4], Jon C Aster[5], Annapoorni Rangarajan[1] and Rajan R Dighe[1]#**

[1]Department of Molecular Reproduction Development and Genetics, Indian Institute of Science Bangalore, Karnataka, India
[2]Molecular Biophysics Unit, Indian Institute of Science Bangalore, Karnataka, India
[3]Department of Pathology, Kidwai Memorial Institute of Oncology, Bangalore, Karnataka, India
[4]Department of Pediatric Oncology, Kidwai Memorial Institute of Oncology, Bangalore, Karnataka, India
[5]Department of Pathology, Brigham & Women's Hospital, Harvard Medical School, Boston, USA
$- SVR and KP equally contributed to the work.

*Running Title: Targeting Notch1 Signaling in Cancer Stem Cells

***Corresponding author:**
Rajan R Dighe, Professor, Department of Molecular Reproduction Development and Genetics, Indian Institute of Science, Bangalore 560012, India, Tel: 9180-2293-2660/3261
Fax: 9180-2360-0999, Email: rdighe@mrdg.iisc.ernet.in

**Supplementary Legends:**

**Supplementary Figure 1: Notch1 mutational analysis in T-ALL patients.**

**Supplementary Figure 2: (A) Effect of MAb 604.107 on LIC subpopulation:** The $CD34^{High}/CD45^{High}$ cells were flow-sorted from the patient derived PBL's followed by culture with control IgG or the MAb 604.107 (10μg/ml) for 72 hours and BrdU incorporation into DNA was determined. (B) Effect of MAb 604.107 on Notch target genes in CCRF-CEM cells.

**Supplementary Figure 3: Effect of MAb604.107 on long-term self-renewing sphere forming cells.** Breast cancer cell lines MCF-7 and MDA-MB-231 cells were allowed to form sphere in semisolid medium in presence of MAb 604.107 for 1 week followed by second generation of sphere formation in the absence of MAb.

**Supplementary Figure 4: Effect of MAb 604.107 on NICD-1, Hes-1, Caspase-3 and BCl-2 expression.** MDA-MB-231 and HCT-116 cells were treated with control of MAb604.107 (20μg/ml) for 48 hours at the end of which whole cell lysate was prepared and level of NICD-1, Hes-1, Caspase-3 and BCl-2 was evaluated in western blot. Beta-actin was used as normalizing control.

**Supplementary Figure 5: Effect of anti-Notch1 antibody treatment on stemness genes in breast and colon cancer cell lines.**

**Supplementary Figure 6: Effect of anti-Notch1 antibody treatment on EMT genes in breast and colon cancer cell lines.**

**Supplementary Figure 7: Effect of MAb604.107 on Dox resistant cell lines.** MDA-MB-231 and HCT-116 cells were cultured in the presence of Dox for three week and Dox-resistant cells lines were established. (A) Expression level of NICD-1 in Dox sensitive and resistant cells. (B) Dox sensitive and resistant MDA-MB-231 and HCT-116 cells were cultured in the presence of control IgG or MAb (20μg/ml) and BrdU incorporation was determined.

**Supplementary Figure 8: Effect of anti-Notch1 antibody treatment on Notch downstream targets and stemness genes in xenografts.**

**Supplementary Figure 9: Expression of NICD-1 in control of MAb treated (A) MDA-MB-231 and (B) HCT-116 xenografts.**

**Supplementary Figure 10: ELISA based epitope mapping of MAb604.107**

**Supplementary Table 1: Phage-display screening for epitope mapping.**

**Supplementary Table 2: Comparison between MAb604.107 with previously reported inhibitory ant-Notch1 Mab.**

# Supplementary Figure-1

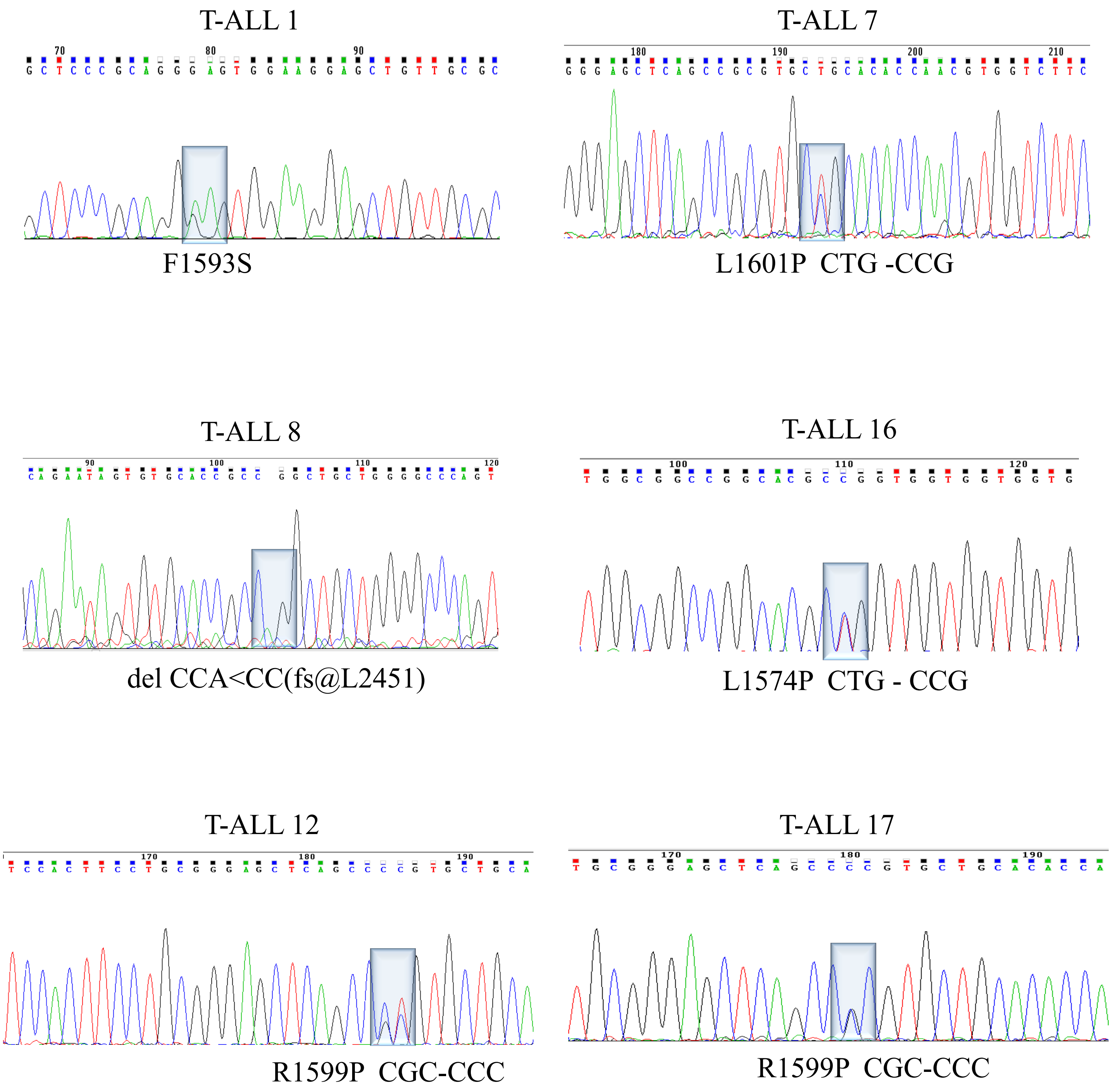

Supplementary Figure-2

(A)

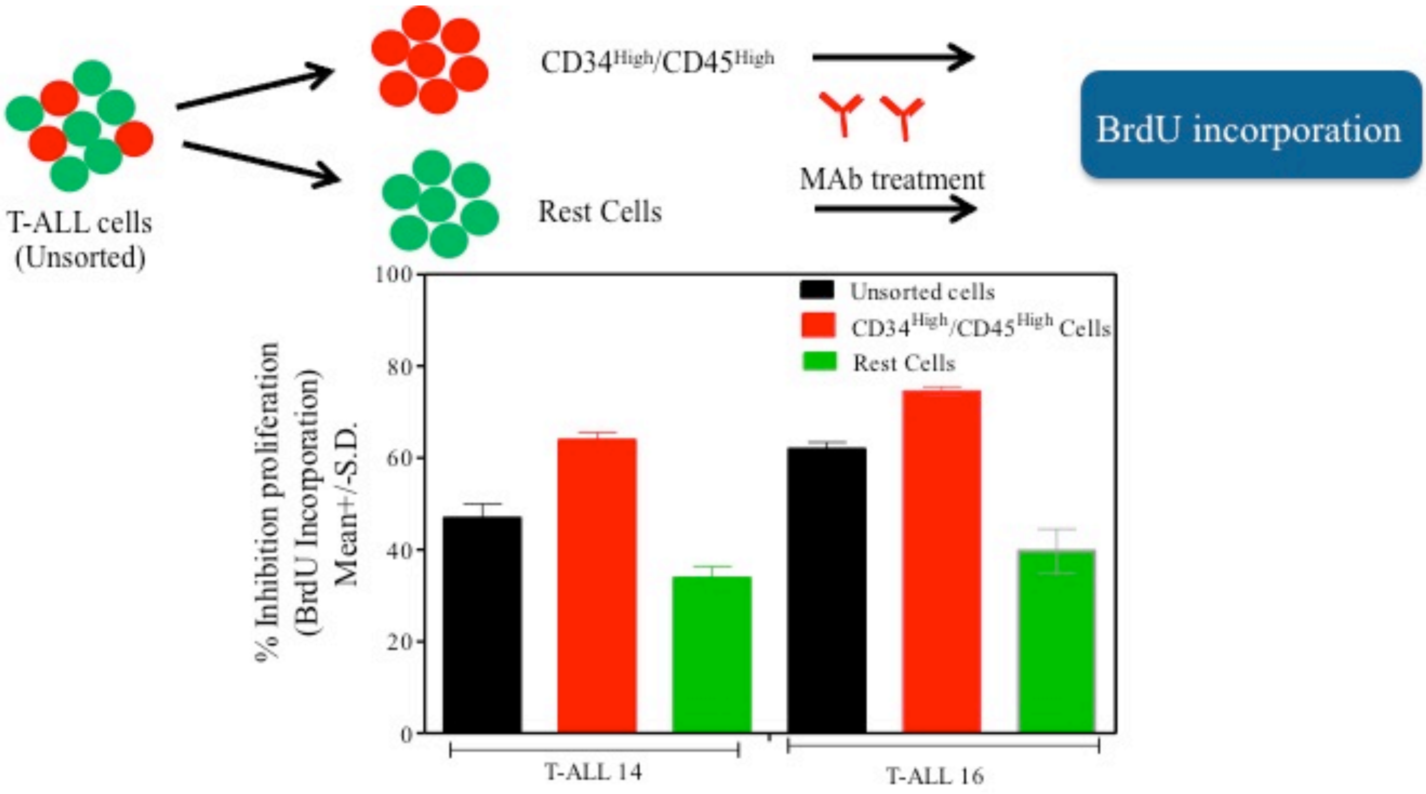


(B)

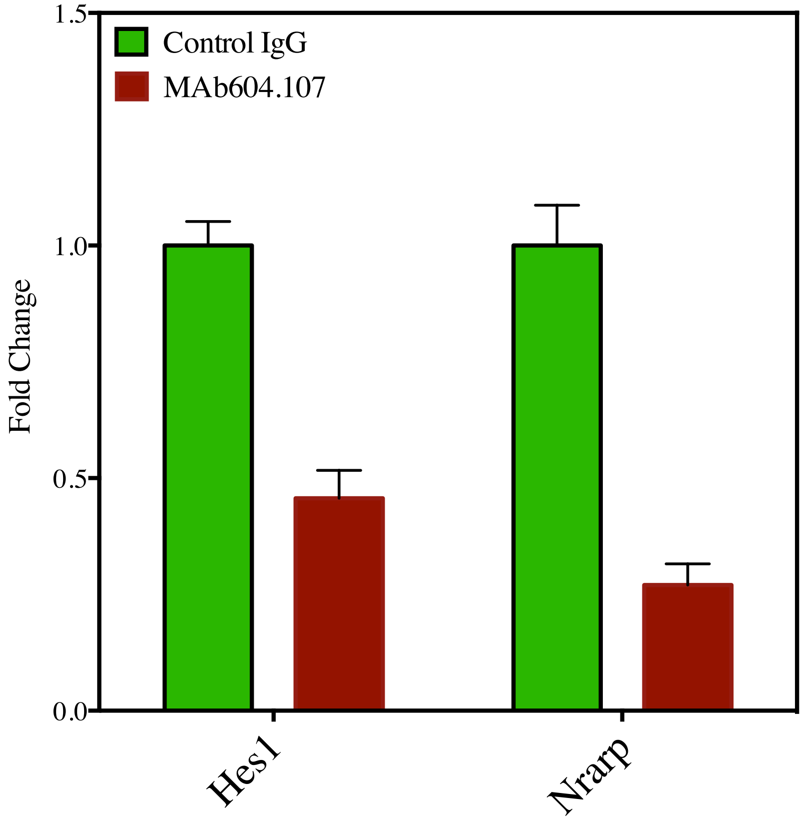

# Supplementary Figure-3

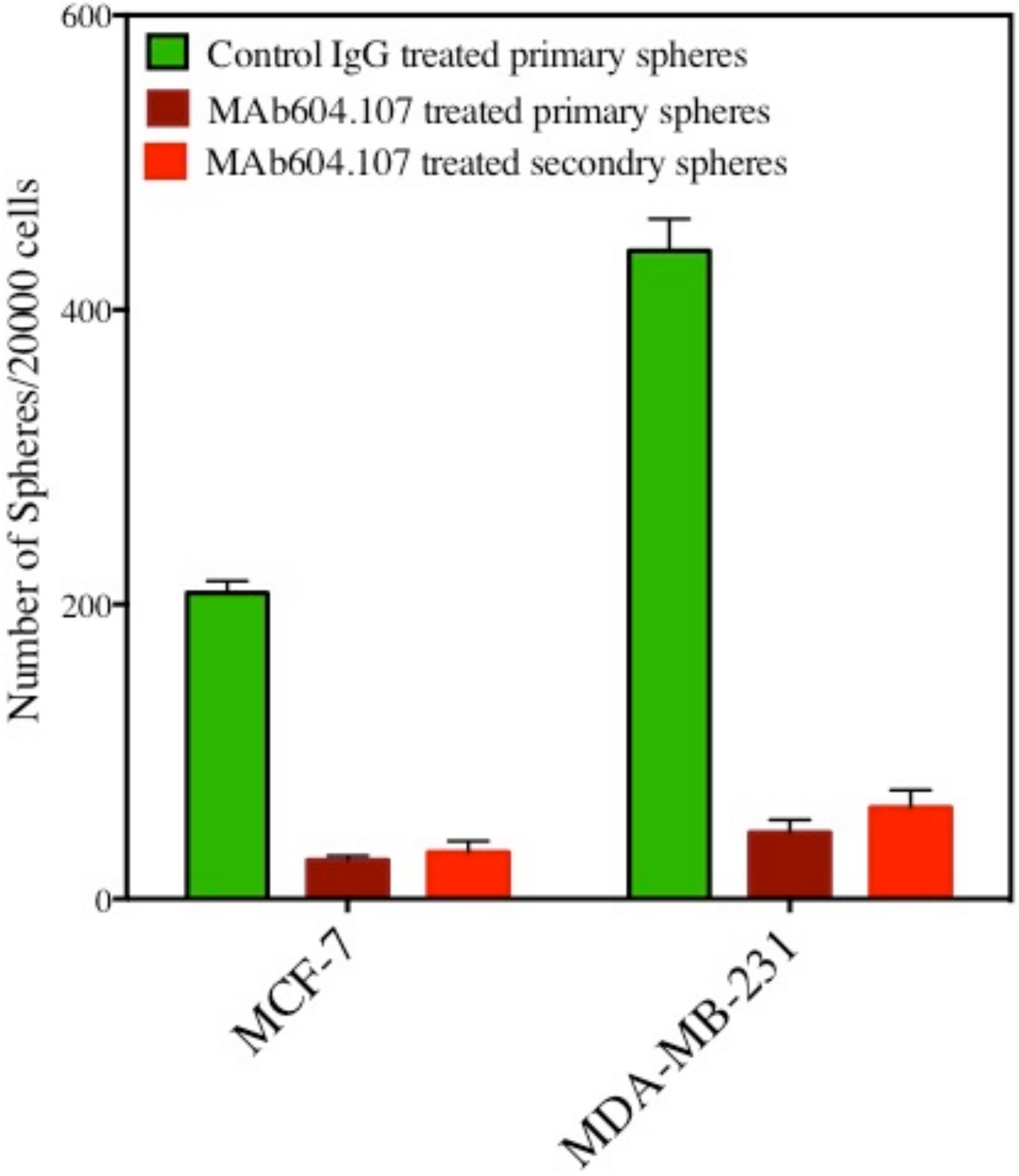

Supplementary Figure-4

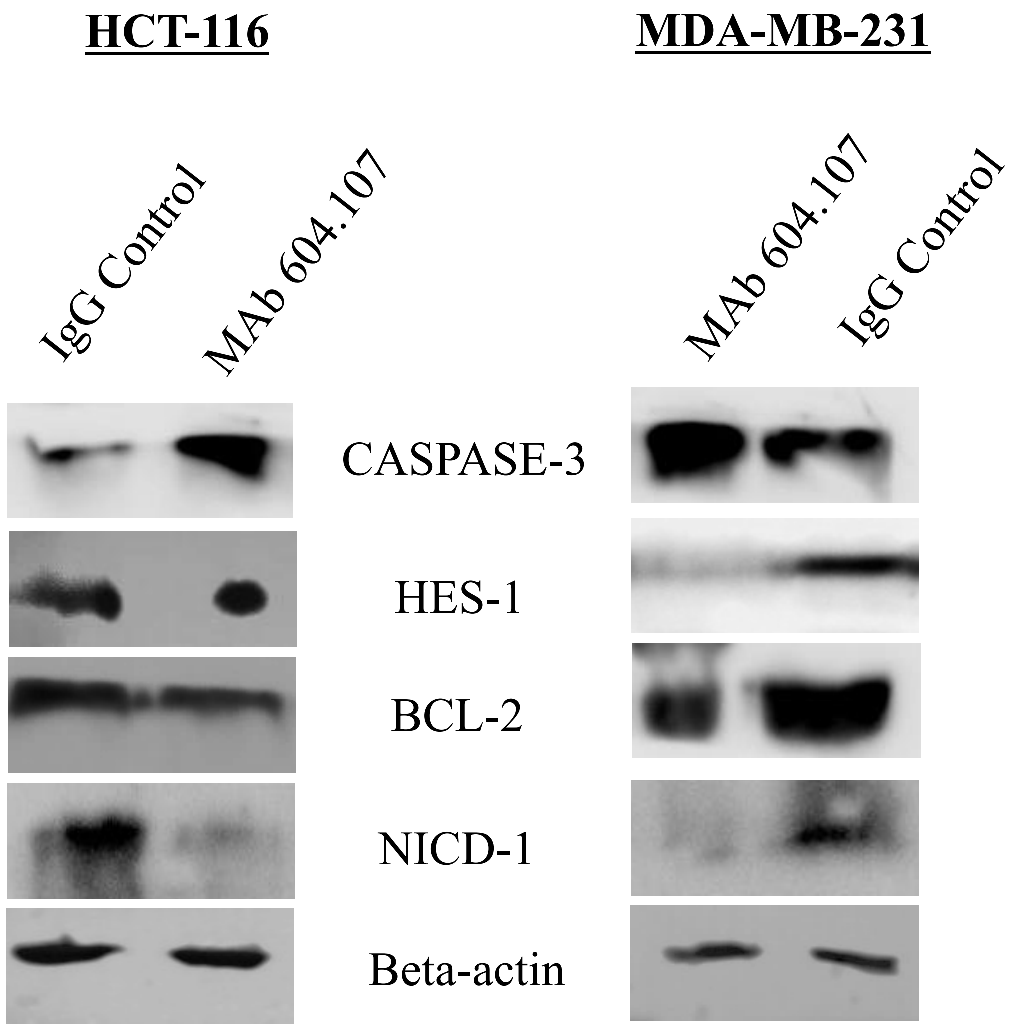

# Supplementary Figure-5

MDA-MB-231

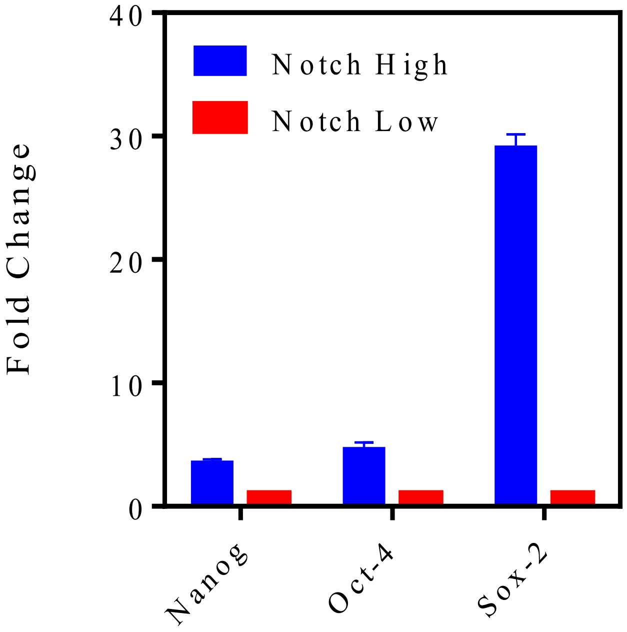


HCT-116

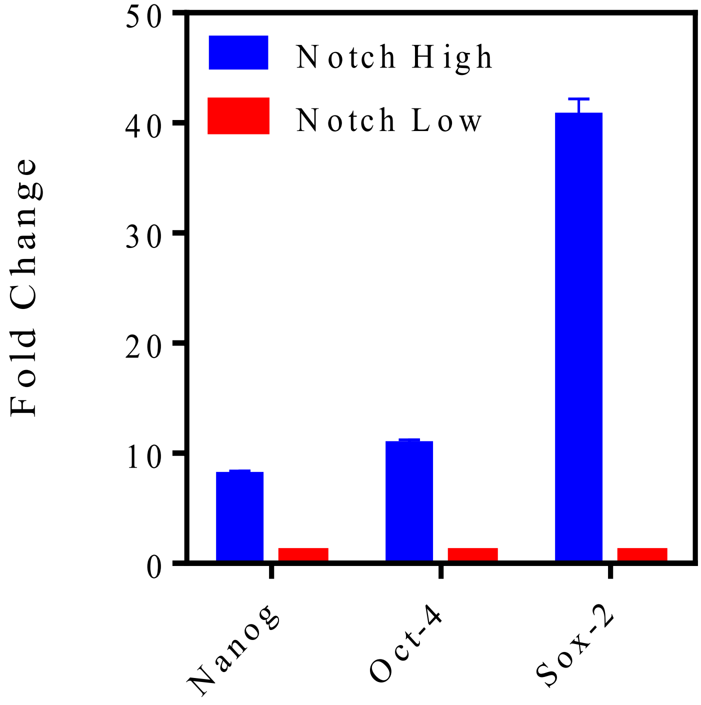

# Supplementary Figure-6

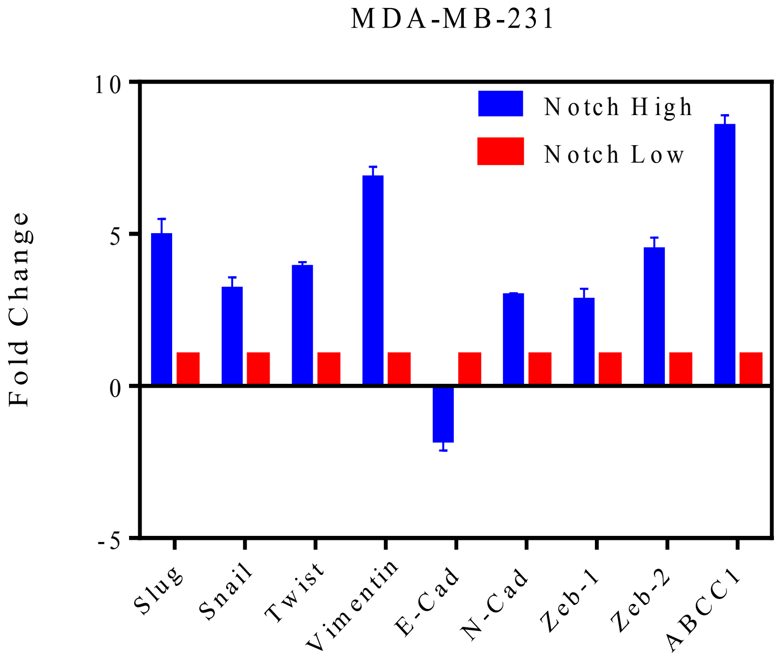


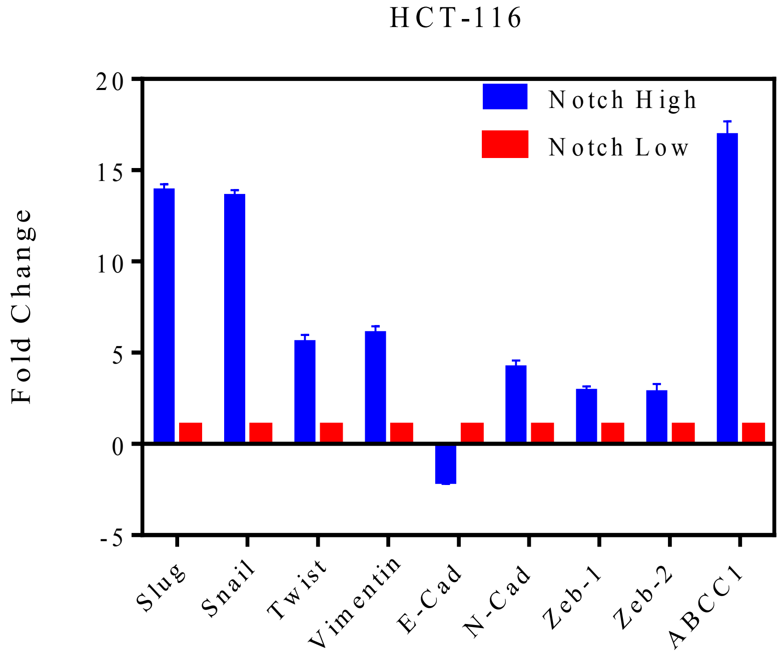

# Supplementary Figure-7

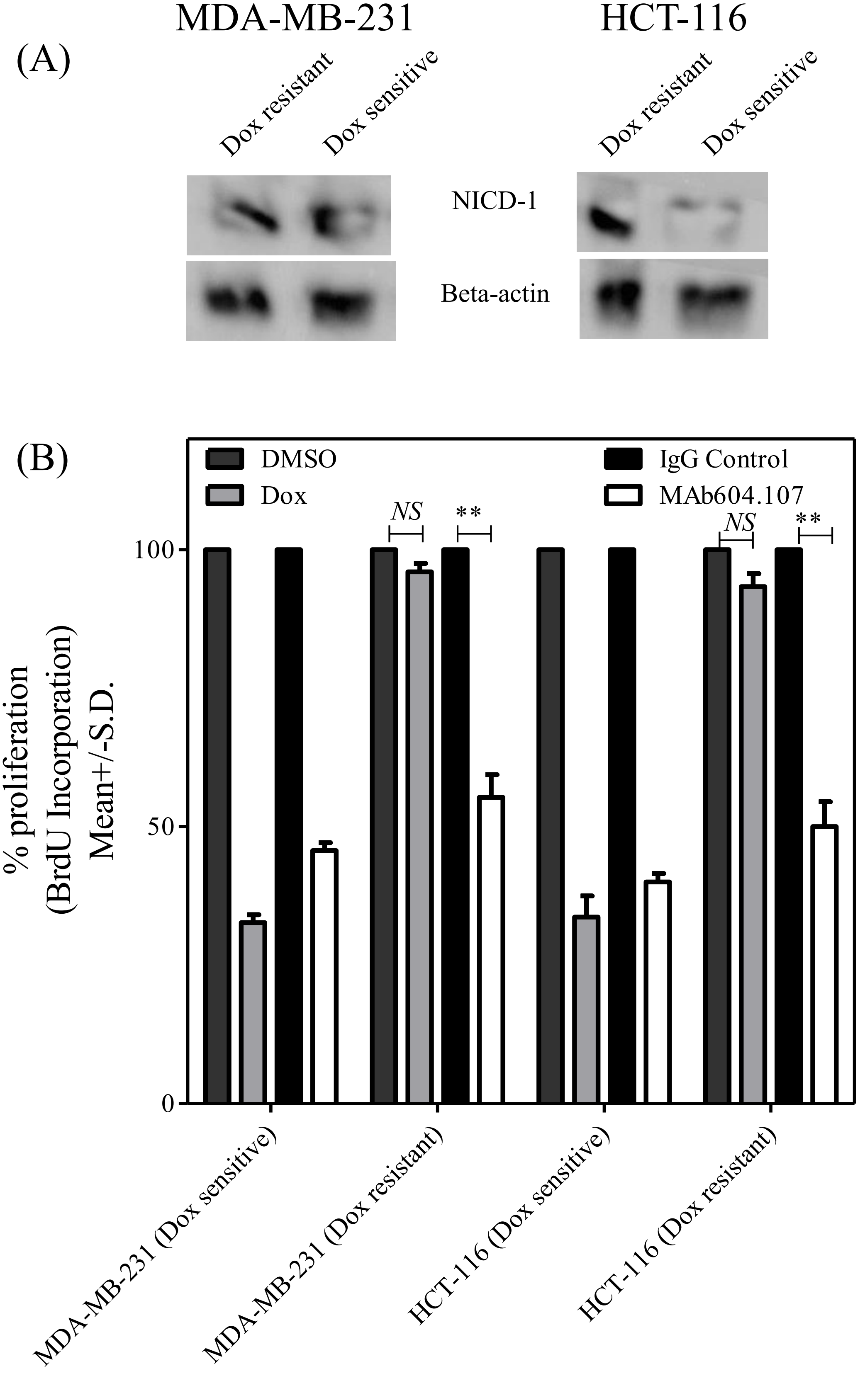

# Supplementary Figure-8

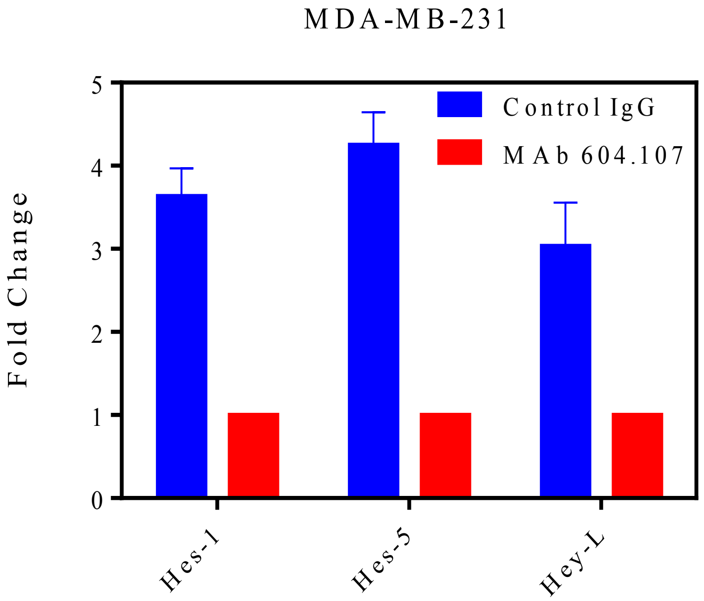


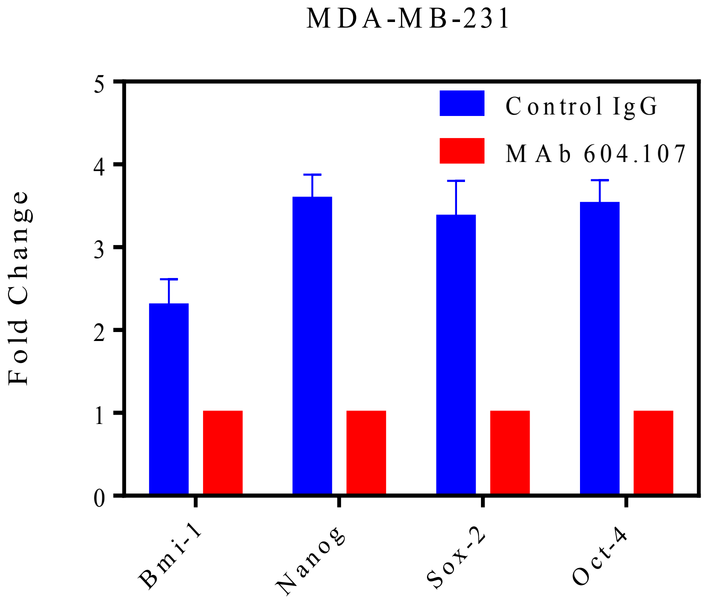


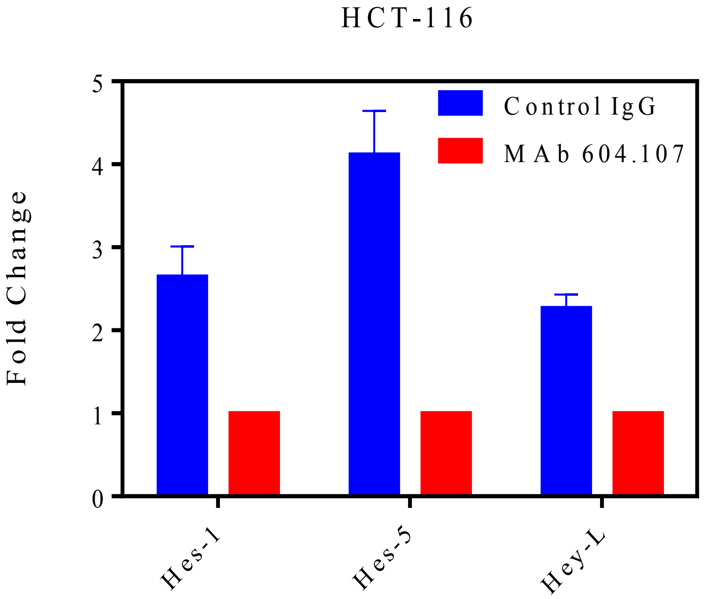


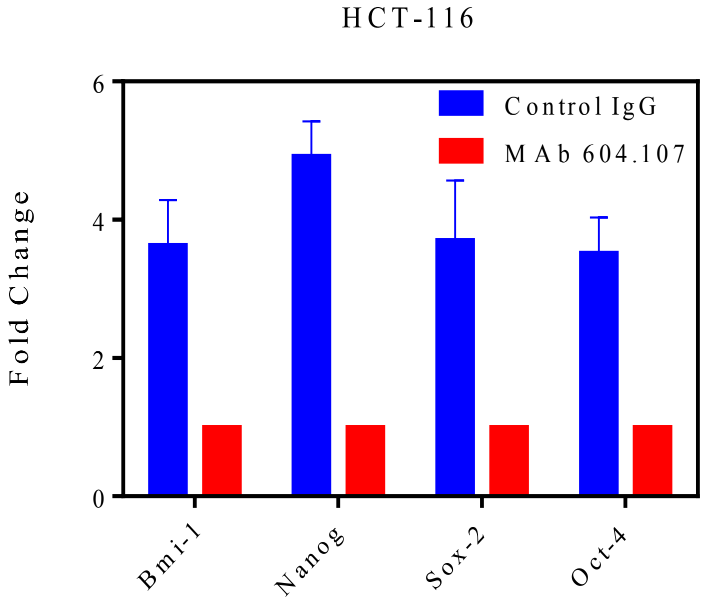

Supplementary Figure-9

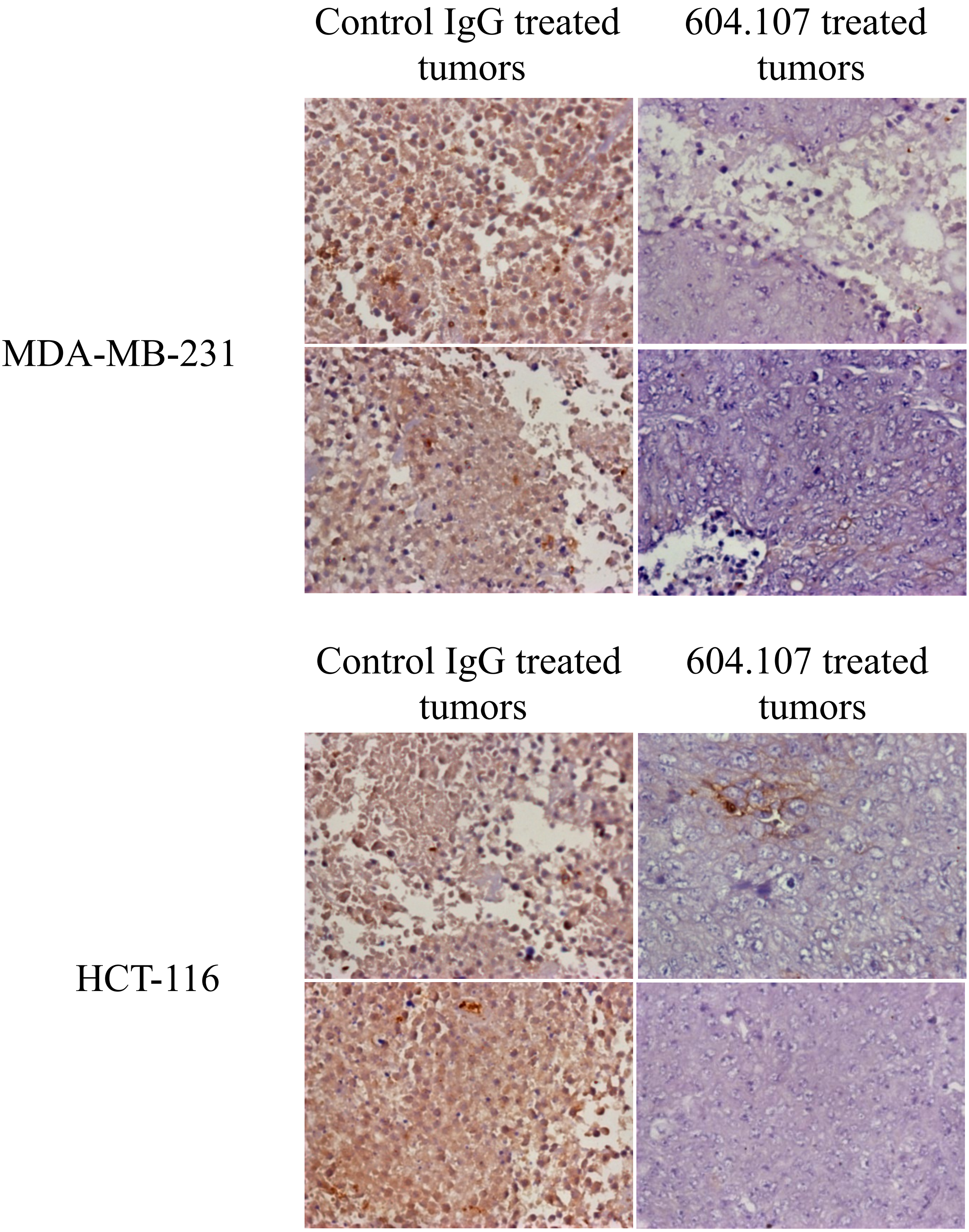

Supplementary Figure-10

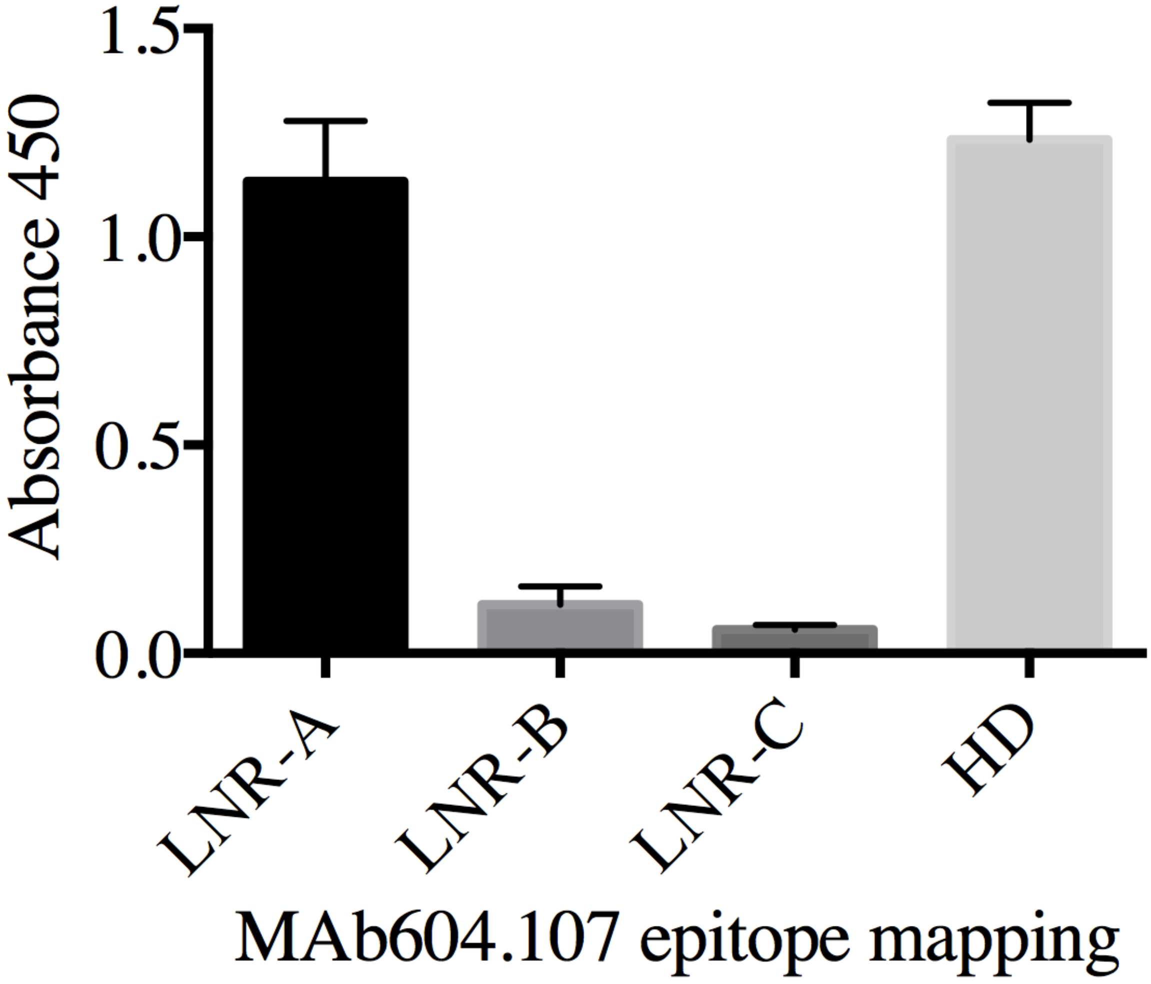

# Supplementary Table-1

| MAb | Putative epitopes in Notch1 NRR domain | NRR domain |
| --- | --- | --- |
| 604.107 | LRNSSFHFLRELSRVL HTNVVFKRD | Heterodimerization (HD) domain |
| 604.107 | SLNFNDPWKNCTQSL QCWKYFS | LNR-A |
| 604.132 | IFPYYGREEELRK | HD |
| 604.132 | SLNFNDPWKNCTQSL QCWKYFS | LNR-B |
| 604.164 | HGQQMIFPYY | HD |

# Supplementary Table-2

| Properties of MAb | Present study 604.107 (Sharma et al.,) | Previous study[18] (Wu et al., Nature 2010) |
|---|---|---|
| Effect on wild-type Notch signaling | Inhibitory (at higher dose) | Inhibitory |
| Affinity for wild-type Notch1 | $5.08x10^{-8}$ | $2.5x10^{-9}$ |
| Effect on mutant Notch1 | Inhibitory (both at higher and lower doses) | Inhibitory |
| Affinity for mutant Notch1 | $5.08x10^{-9}$ | ND |
| Specificity for mutant Notch1 | At lower concentration specific for mutant Notch1 without affecting wild-type signaling | ND |
| Effect on LIC subpopulation | Deplete LIC subpopulation | ND |
| Impact on CSC and chemoresistance cells | Irreversibly inhibit CSCs | ND |

ND- not determined
